# Of yeast, mice and men: MAMs come in two flavors

**DOI:** 10.1186/s13062-017-0174-5

**Published:** 2017-01-25

**Authors:** Maria Sol Herrera-Cruz, Thomas Simmen

**Affiliations:** Department of Cell Biology, Faculty of Medicine and Dentistry, University of Alberta, Edmonton, Alberta T6G2H7 Canada

**Keywords:** Mitochondria-associated membrane, MAM, Mitochondria-ER contacts, MERCs, Human, S. cerevisiae, Yeast

## Abstract

The past decade has seen dramatic progress in our understanding of membrane contact sites (MCS). Important examples of these are endoplasmic reticulum (ER)-mitochondria contact sites. ER-mitochondria contacts have originally been discovered in mammalian tissue, where they have been designated as mitochondria-associated membranes (MAMs). It is also in this model system, where the first critical MAM proteins have been identified, including MAM tethering regulators such as phospho-furin acidic cluster sorting protein 2 (PACS-2) and mitofusin-2. However, the past decade has seen the discovery of the MAM also in the powerful yeast model system *Saccharomyces cerevisiae*. This has led to the discovery of novel MAM tethers such as the yeast ER-mitochondria encounter structure (ERMES), absent in the mammalian system, but whose regulators Gem1 and Lam6 are conserved. While MAMs, sometimes referred to as mitochondria-ER contacts (MERCs), regulate lipid metabolism, Ca^2+^ signaling, bioenergetics, inflammation, autophagy and apoptosis, not all of these functions exist in both systems or operate differently. This biological difference has led to puzzling discrepancies on findings obtained in yeast or mammalian cells at the moment. Our review aims to shed some light onto mechanistic differences between yeast and mammalian MAM and their underlying causes.

**Reviewers**: This article was reviewed by Paola Pizzo (nominated by Luca Pellegrini), Maya Schuldiner and György Szabadkai (nominated by Luca Pellegrini).

## Background

Endoplasmic reticulum (ER)-mitochondria contacts were described for the first time by Wilhelm Bernhard on electron micrographs of rat liver in 1952 [1] and 1956 [2, 3]. However, it was not until their first biochemical isolation in 1990 that their significance for membrane contact site (MCS) research became clear. In her landmark paper, Jean Vance demonstrated for the first time that the endoplasmic reticulum (ER) must make physical contacts with mitochondria to allow for proper lipid synthesis [4]. Vance subsequently coined the term mitochondria-associated membrane (MAM, originally called fraction X) in a follow-up paper [5]. The MAM is thought to be contiguous with the remainder of the ER, but physically attached to mitochondria and thus biochemically distinct from pure ER or pure mitochondria. Vance determined that enzymes catalyzing phosphatidylserine (PS), phosphatidylethanolamine (PE) and phosphatidylcholine (PC) synthesis localize to ER-mitochondria contact sites that have recently been proposed to be called mitochondria-ER contacts (MERCs, [5–7]). Central to this lipid metabolism found on the MAM is the transfer of PS from the ER to mitochondria, followed by its enzymatic transformation to PE inside mitochondria [8].

In liver and other mammalian cells and cell lines, the distance between the ER and mitochondria in contact sites typically measures 15–30 nm under resting conditions [7]. However, cell stress can transform ER-mitochondria contacts into a much tighter version of 10 nm via mechanisms that are currently not fully understood [7, 9]. While MAM research progressed rapidly using mammalian model systems, MAMs could not be isolated in the yeast model system for a long time [10], even though ER and mitochondria were seen apposed on early electron micrographs of the yeast *Saccharomyces cerevisiae* [11]. Another reason for this delay was the limited apposition between the ER and mitochondria that yeast researchers detected in their model system, relative to the more frequent contacts they saw between the ER and lipid droplets [12].

Nevertheless, we know today that the MAM is but one of many MCS that exist inside yeast and mammalian cells [13]. The palette of MAM functions has been dramatically expanded over the past decade and now includes the exchange of lipids, ions and second messengers [14], mitochondrial fission [15], and the induction of autophagy [16]. Despite a partial overlap of MAM functions in yeast *S. cerevisiae* and mammalian cells, important differences exist between the two model systems that often cause confusion to researchers (summarized in Table 1). This review aims to list common properties and differences, as we know them today, to help the community sort out how to best use either mammalian or yeast models to answer MAM-specific questions.

**Table 1:** Current overview of MAM functions and comparison of how they operate in mammalian and yeast model systems

Function (including brief description)	Mammalian-specific characteristics	Yeast-specific characteristics
Phosphatidylserine (PS) Transfer: PS is made on the ER, but transferred to mitochondria for the production of phosphatidylethanolamine (PE) [4, 84]	PS transfer occurs on a triple contact site between ER, OMM and IMM [25–27], requires ATP [90] and cytosolic Ca^2+^ [91].	PS transfer occurs at ER mitochondria contact sites [96], but does not require ATP [17, 97]. PS transfer is not obligatory, since Psd2p can replace mitochondrial PE production [98, 99].
Role of sterols on MAM	MAM has lipid raft characteristics and is marked with caveolin [116–122]. ORP5 and ORP8 form a protein bridge with mitochondrial PTPIP51 [130].	No raft characteristics known as of today [123].
Ca^2+^ handling at the MAM; mitochondria receive Ca^2+^ from the ER upon formation of a Ca^2+^ microdomain [155–163]	Mitochondria import Ca^2+^ via the mitochondrial Ca^2+^ uniporter (MCU) [148–150]. Ca^2+^ is required for mitochondrial dehydrogenases [132] and respiration [133].	No MCU present [148–150]. Ca^2+^ is required for mitochondrial dehydrogenases [151]; increase of Ca^2+^ in the cytoplasm boosts mitochondrial respiration [194].
MAM Ca^2+^ signaling in apoptosis	Massive MAM Ca^2+^ transfer accelerates apoptosis [39, 192].	Ca^2+^ is released from the ER [193].
ER chaperones on the MAM	ER chaperones on the MAM control MAM Ca^2+^ transfer and cytosolic Ca^2+^ waves [180–188].	None detected.
Currently known MAM tethers or proteins regulating MAM tethering	PACS-2 [38, 40], mitofusin-2 [42–45], BAP31/Fis1 (ARCosome) [41], IP3R/VDAC/Grp75 [73], PTPIP51/VAPB [74–76] PERK [65, 66]	ERMES [18–20], EMC [34]
MAM and mitochondrial fission	Drp1 oligomerizes on MAM to mediate mitochondria fission [15]	Drp1 oligomerizes on MAM to mediate mitochondria fission [15]
MAM as point of origin for autophagy	MAM is material for isolation membrane [16]. Implicated MAM proteins are calnexin, Drp1, FUNDC1 [220], Rab32, syntaxin-17 [222, 223], PACS-2, mitofusin-2 [16]	ERMES mutants show no defect in Atg8p recruitment, but are defective in mitophagy and lipid supply for phagophore formation [22, 224]

### Proteins mediating formation of the MAM

**Fig. 1:**
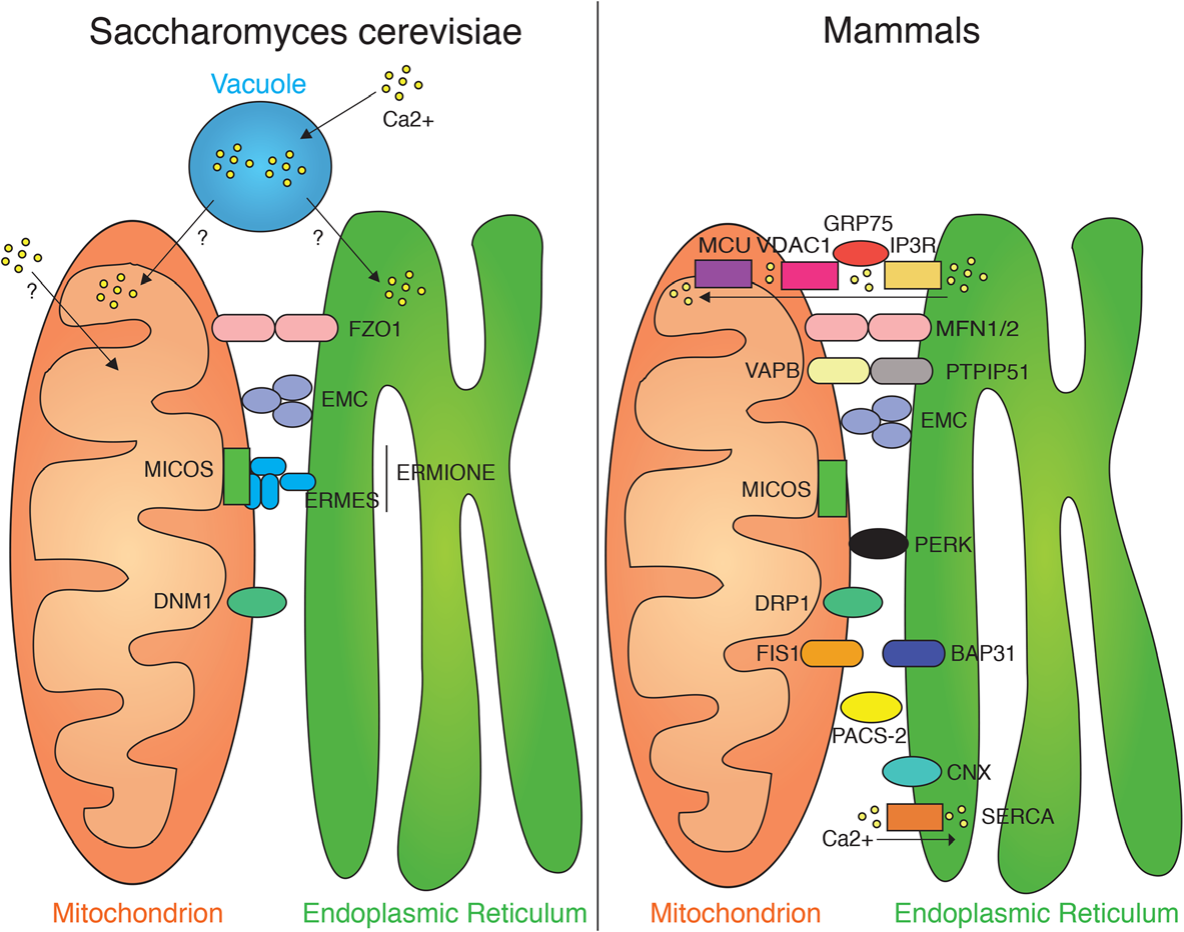
Overview of ER-mitochondria tethers (and their regulators), as well as Ca^2+^ flux in yeast and mammalian cells

Central to our understanding of the MAM structure are the proteins required for its formation. The discovery of these proteinaceous tethers was initially based on the observation that proteinases detach the ER from mitochondria in both mammalian and yeast model systems [9, 17]. As we will describe later on, ER-mitochondria contacts have a distinct functional significance for yeast and mammalian ER-mitochondria crosstalk. These differences are partially mirrored in our progress to characterize ER-mitochondria tethers. While the *S. cerevisiae* model has taken advantage of genetic screening power that has led to the identification of two tethering complexes so far, mammalian systems currently benefit from a larger array of functional readouts of the contacts (a summary of the main proteins involved in ER-mitochondria tethering in yeast and mammalian cells is shown in Fig. 1).

As one of the best-characterized structures, the ER-mitochondria encounter structure (ERMES) was identified in a mutagenesis screen involving an artificial ER-mitochondria tether in *S. cerevisiae* [18]. ERMES contains the ER transmembrane protein Mmm1p and the cytosolic Mdm12p in a complex with two outer mitochondrial membrane (OMM) proteins, Mdm34p and Mdm10p [19]. Gem1p, a Ca^2+^-binding, rho-like GTPase and ortholog of mammalian MIRO-2, is a negative ERMES regulatory protein [20]. In addition to mediating ER-mitochondria tethering, ERMES also influences the distribution of mitochondria during cell division [21], as well as the recognition of mitochondria for autophagy [22]. ERMES interacts genetically with the mitochondrial contact site complex (MICOS). With ERMES, this hexameric complex forms the ER-mitochondria organizing network (ERMIONE), which contains additional components involved in the import of mitochondrial proteins (TOM complex) and the sorting and assembly machinery (SAM) of the OMM [23, 24]. ERMIONE likely forms ER-proximal cristae junctions identified in early MAM research as critical for PS trafficking [25–27]. From this role, it is no surprise that MICOS is an important determinant of mitochondrial cristae formation, and mitochondria metabolism [28]. MICOS also actively participates in lipid metabolism, as it determines PE formation [29]. Despite important roles for ERMES in yeast and the formation of ERMIONE, present also in mammalian cells, ERMES is absent in metazoa [30]. Curiously, this is not the case for MICOS [31–33], as well as ERMES-regulatory proteins, given the conservation of the inhibitory Gem1 (MIRO-2) [20] and the activating Lam6 (not characterized in mammalian systems at this point).

A more recently identified tethering complex in *S. cerevisiae* is the ER membrane protein complex (EMC). In yeast, EMC is a heteromeric hexamer that contains Emc1p, Emc2p, Emc3p, Emc4p, Emc5p and Emc6p [34]. Yeast EMC plays a role for the import of PS into mitochondria, but this could be direct or indirect, while its role for ER-mitochondria Ca^2+^ signaling is currently unknown. Consistent with its high level of conservation during evolution, mammalian cells also contain EMC, but a version with 4 extra proteins, Emc7, Emc8, Emc9 and Emc10 [35]. Here, in addition to tethering mitochondria to the ER, EMC also acts as a chaperone for the assembly of multipass transmembrane proteins [36]. Further research will have to determine which of these functions is the main role of EMC.

None of the currently known mammalian tethering molecules have been identified via genetic screening. Instead, researchers frequently used empiric approaches, often based on the quasi-synaptic ER-mitochondria Ca^2+^ flux [37]. The first example of these is the phosphofurin acidic cluster sorting protein 2 (PACS-2), the first discovered MAM-regulatory protein [38], which is not present in yeast. PACS-2 knockdown detaches the ER from mitochondria [38]. As expected from a protein involved in MAM tethering, PACS-2 is needed for proper apoptosis progression that normally occurs following the transfer of massive amounts of Ca^2+^ from the ER to mitochondria [39]. The kinase Akt activates PACS-2 by phosphorylating it on serine 437, a prerequisite to maintain MAM formation and Ca^2+^ availability for mitochondria [40]. PACS-2 also controls the proteolytic cleavage of BAP31 (also known as BCAP31), and thus influences directly a tethering complex between BAP31 and mitochondrial Fis1 called the ARCosome [41]. While yeast expresses a BAP31-like protein (Yet3p), no PACS-2 resembling sequences exist in this organism (unpublished observations). In contrast, Fis1 is found in yeast, but it is currently unclear whether it can interact with Yet3p and form a tether here as well.

Similar to PACS-2, the reduction of ER-mitochondria apposition in a knockout (ko) cell model led to the discovery of mitofusin-2 as a tethering factor [42]. Consistent with this role, we have found that mitofusin-2 ko cells lack fluorescence derived from an ER-mitochondria dimeric split green fluorescent protein that is based on reconstitution of the calnexin-TOM20 interaction when MAMs are formed [43]. Like PACS-2 knockdown cells, mitofusin-2 ko cells are resistant to apoptosis during ER stress [44], but also to cardiomyocyte death induced by hypoxia and H_2_O_2_ [45]. Conversely, cancer cells over-expressing mitofusin-2 show accelerated apoptosis, accompanied by increased ER-mitochondria Ca^2+^ flux [46–48]. PACS-2 and mitofusin-2 share an induction of ER stress in their absence [38, 49], leading to abnormal expansion of the ER in the case of mitofusin-2 ablation [42]. While these results provide strong evidence that mitofusin-2 promotes ER-mitochondria tethering, the exact role of mitofusin-2 for MAM maintenance has been challenged by several studies suggesting mitofusin-2 is an antagonist for MAM formation. Specifically, when analyzing the amounts of ER-mitochondria apposition of less than 15–20 nm, they show ER-mitochondria contacts increase upon mitofusin-2 depletion [50–53]. Moreover, these studies demonstrate that this consequence of mitofusin-2 depletion is not further increased in the presence of presenilin-2 mutant protein [54] that normally increases ER-mitochondria tethering in wild type cells due to a relative shift of Ca^2+^ content from the ER to mitochondria [55]. Together, these observations could explain why certain apoptosis pathways proceed faster in mitofusin-2 depleted cells, including ceramide-induced apoptosis [51], and doxorubicin-induced apoptosis [56]. Furthermore, the absence of mitofusin-2 inconsistently alters mitochondrial respiration capacity in a variety of model systems [53, 57–59].

How can these drastically discrepant findings be explained? In our opinion, these observations are likely only apparently contradictory and they could be based on incomplete characterizations of mitofusin-2 ko or knockdown cells in some studies. *First*, the Pellegrini [7] and Nabi labs have recently demonstrated that ER subtypes form distinct contacts with mitochondria: while smooth ER (sER) is apposed at 10 nm with mitochondria (tight), rough ER (rER) localizes at a 50 nm (loose) distance with mitochondria [60]. Interestingly, the ratio of these MAM subtypes is under the control of mitofusins in fibrosarcoma cells. Here, knockdown of the ubiquitin ligase Gp78 can decrease overall MAM formation and rER-mitochondria apposition, due to increased amounts of both mitofusin-1 and mitofusin-2, normally degraded by the proteasome via Gp78 [60, 61]. In this system, depletion of single mitofusins had no effect on the overall amount of MAMs. However, the knockdown of mitofusin-1 (but not of mitofusin-2) was able to increase sER-based MAM relative to rER-based MAM in the presence of mitofusin-2. This suggests that mitofusin-1 normally acts to prevent excessive amounts of tight sER-mitochondria contacts [60]. In contrast, mitofusin-2 repressed loose rER-mitochondria contact formation in this system. Together, these results clearly show that mitofusins act in concert to reduce MAM overall, and that the respective expression levels of mitofusins determines the ratio of sER/rER-mitochondria contact formation. However, in our opinion, they do not shift the balance significantly towards either hypothesis on the global role of mitofusin-2 for MAMs, since mitofusin-2 knockdown alone had no measurable effect on MAMs in this system.

*Second*, varying extents of adaptation could explain discrepant findings on the role of mitofusin-2. Unequivocally, the absence of mitofusin-2 alters intracellular Ca^2+^ handling (see below, Fig. 1), since in these cells, there is an increased Ca^2+^ content within the ER [42, 52]. This increased ER Ca^2+^ content results in mitochondria taking up more Ca^2+^ upon the addition of equal amounts of inositol 1,4,5-trisphosphate receptors (IP_3_R) agonist [62], but not if correcting for these differences by adding agonist so to release equal amounts of Ca^2+^ into the cytosol [42, 52]. Moreover, mitofusin-2 depletion results in compensatory mechanisms in a cell culture-dependent manner [52], for instance by the initiation of ER stress [44] or the downregulation of the MCU, observed to varying extents [52] in Mfn2−/− cells [51], but not in cells, where mitofusin-2 has been knocked down [51]. Both observations are critical in our further investigation of the role of mitofusin-2: the downregulation of MCU likely serves to prevent Ca^2+^ overload due to the hyperresponsive ER in mitofusin-2 depleted cells.

The triggering of the ER stress response could lead to the *third* and potentially most important reason that different labs came to different conclusions about mitofusin-2. Several labs, including ours, have shown that ER stress leads to secondary formation of tight ER-mitochondria contacts, which alter the global cellular Ca^2+^ handling at the ER-mitochondria interface [9, 63, 64], but also within the cytosol, even when the cytosolic Ca^2+^ response is kept even [51]. Of particular interest in this context is PERK, known to be tightly associated with the Mfn2−/− phenotype [44], but also the *de novo* formation of stress-dependent contacts [65, 66]. Future studies involving more detailed characterizations of intracellular Ca^2+^ handling and the ER stress phenotype will have to untangle the equally important roles of mitofusin-2 in ER-mitochondria tethering and in the prevention of ER stress.

Yeast *S. cerevisiae* encodes Fzo1p, a protein that is the mitofusin-1 and mitofusin-2 homolog [67, 68]. Like mammalian mitofusins, Fzo1p localizes to the OMM and mediates mitochondrial fusion. Here, Fzo1p interacts with MICOS [69]. Yeast deleted of *FZO1* do not grow well on fermentable carbon sources and exhibit a petite phenotype, which would be expected if it were a tether; alternatively, this property could depend solely on its role in mitochondrial fusion, two hypotheses to be tested in the future. Some interesting parallels exist between ubiquitination of mitofusin-2 and Fzo1p: mammalian Gp78 ubiquitinates both mitofusins and thus controls the ratio of MAMs made from rER and sER [60]. Similar to Gp78, yeast Mdm30p ubiquitinates Fzo1p to eliminate this GTPase once mitochondrial fusion has taken place [70]. Other potential connections might exist with the yeast ubiquitin ligase Met30p [71], a factor promoting ER-mitochondria interaction, as well as mammalian MITOL [72].

A less characterized tethering system is based on the formation of a protein complex between IP_3_Rs, VDAC and the outer mitochondrial membrane chaperone Grp75 [73]. Whether this complex is required for ER-mitochondria tethering is currently unclear, since no obvious differences in ER-mitochondria apposition result in IP_3_R triple knockout cells [9]. Another tethering complex can form between the OMM protein PTPIP51 and the ER vesicle-associated membrane protein- associated protein B (VAPB), an integral membrane protein. As typical with any mammalian MAM tether, its disruption also causes impaired mitochondrial Ca^2+^ import [74]. Recent studies have identified TDP-43 [75] and fused in sarcoma (FUS) as inhibitors of the PTPIP51-VAPB complex [76]. No information regarding these tethering complexes is available from yeast.

### Lipid and sterol metabolism: discovery of the MAM in liver cells and parallels in the yeast system

**Fig. 2:**
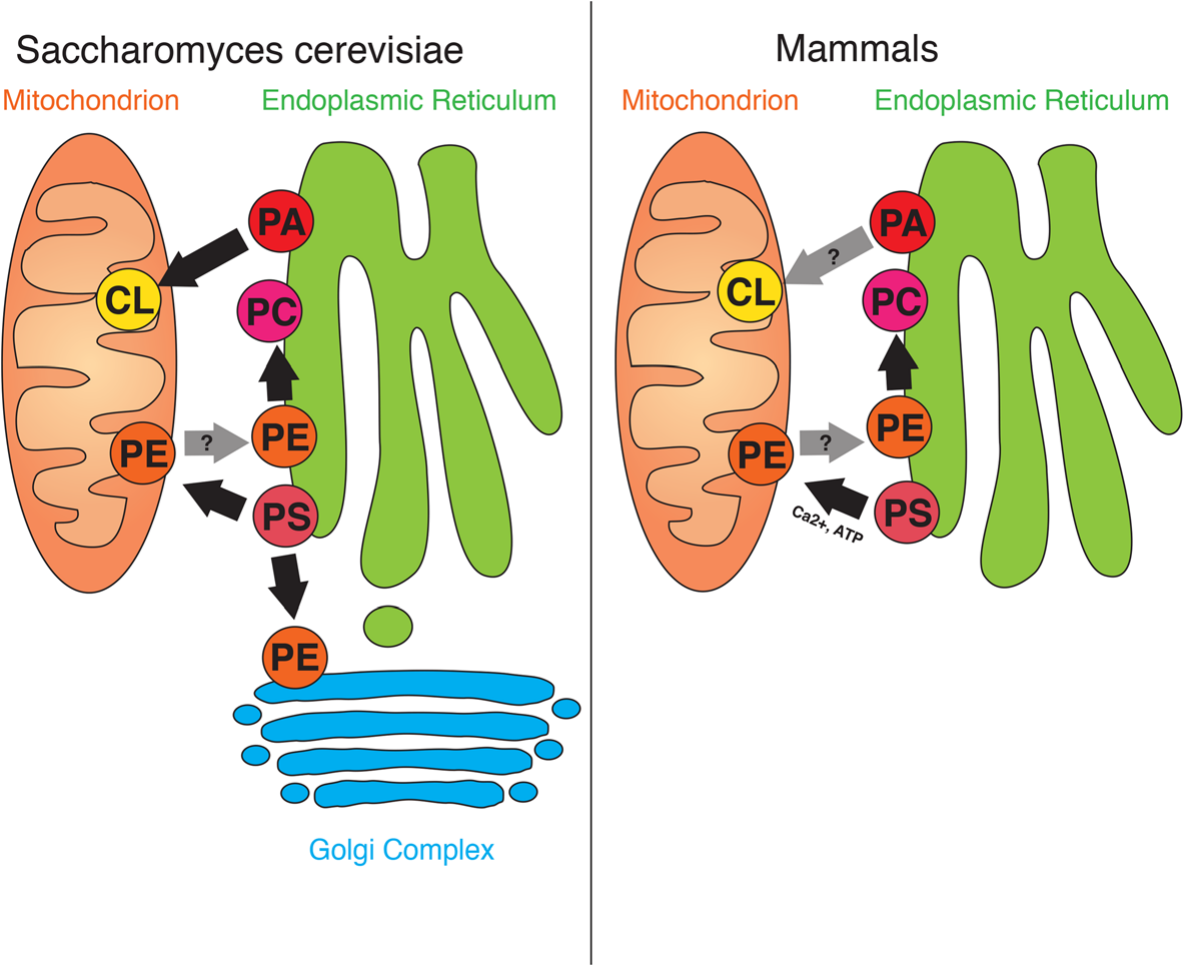
Overview of ER-mitochondria lipid flux in yeast and mammalian cells. The location of synthesis for phosphatidylserine (PS), phosphatidylethanolamine (PE), phosphatidylcholine (PC), phosphatidic acid (PA) and cardiolipin (CL) are shown. Transport pathways (known: *black*; suspected: *grey*), as well as their respective ATP and Ca^2+^ requirements are indicated

Central to the original identification of the MAM is intracellular lipid transport (Fig. 2). Research from the 1970s had demonstrated that mitochondrial PS is imported from microsomes to mitochondria in the liver [77–79]. This was later reproduced in mammalian cell culture models, such as BHK-21 cells [80]. However, in both yeast and mammalian systems, researchers of the period hypothesized at the time that PS uses proteinaceous shuttles to transfer from the ER to mitochondria [81]. The characterization of any mechanism was complicated by the fact that mitochondria could initially not be biochemically separated from associated ER membranes in yeast, leading to the erroneous claim that yeast mitochondria can synthesize PS [82].

In mammalian liver in contrast, this biochemical separation was easier to achieve [83], leading to the landmark discovery that PS is made on the ER-derived MAM, from where it is transported straight to mitochondria to become decarboxylated to yield PE using MAM/MERCs [4, 84]. While little information is available about the transport of PE from the mitochondria to the ER [8], the MAM is known to accommodate PS transfer on a triple contact site, formed with ER apposed to outer and inner mitochondrial membranes (OMM, IMM) [25, 26]. While this lipid transfer could technically use vesicular transport similar to the recently discovered mitochondria-derived vesicles [85], research has so far provided evidence only for direct transfer at apposition sites [84, 86, 87].

In the liver, the localization of lipid metabolic enzymes has been confirmed not only via biochemical techniques, but also via EM [5]. It is currently unknown how these enzymes localize to the MAM, but some might use sequences that lead to their association with mitochondrial membranes, like acyl-CoA:diacylglycerol acyltransferase 2 [88]. Some of these lipid-modifying proteins could themselves serve as ER-mitochondria tethers, a hypothesis that is supported by results showing that the ER-mitochondria transfer of newly synthesized lipids occurs always faster than the one of pre-existing lipids [87]. Another interesting observation is that not only lipid synthesis [89], but also inter-organellar PS transfer requires ATP [90] and cytosolic Ca^2+^ [91] in mammalian systems. However, ATP is neither needed when the cytosolic Ca^2+^ concentration is raised 1000-fold [91], nor in reconstituted in vitro systems [92]. These findings propose a role of cytosolic Ca^2+^ and Ca^2+^ microdomains for MAM formation (see next chapter). They also identify the need of an ATP-consuming mechanism of currently unknown identity for the maintenance of MAM in intact mammalian liver cells [93].

In the yeast *S. cerevisiae* model system, the laboratory of Günther Daum started resolving the confusion regarding the localization of lipid metabolizing enzymes [94]. In their studies, PS synthase was found associated with the *S. cerevisiae* ER rather than mitochondria [95], which then led to the discovery of a shuttling mechanism for PS and PE between the ER and mitochondria in yeast [96]. Unlike the mammalian liver cell model, yeast lipid shuttling at the MAM, formed by around 100 individual membrane contact sites per yeast cell [17], does not require ATP [17, 97]. This is a peculiar difference between the yeast and mammalian systems that has important implications for MAM experiments. Any experiment using mammalian cells must allow for the production of sufficient, preferably mitochondrial ATP, whereas such a requirement does not exist for yeast.

Further, marked differences in lipid production are recognized between the mammalian and the yeast system. Importantly, although yeast can also shuttle PS from the ER to mitochondria, where it is decarboxylated to yield PE, this is not an obligatory step, unlike in mammalian liver cells. This is explained by the fact that the decarboxylase activity on PS is not exclusively localized to mitochondria in the yeast model system. Instead, while Psd1p localizes to mitochondria, its sister protein Psd2p is found on the Golgi complex and the vacuole, thus exhibiting striking differences of this mechanism to mammalian cells [98, 99]. Following translocation of PE by largely unknown mechanisms, this lipid undergoes transfer of a methyl group by MAM-localized PE methyltransferase (PEMT) in mammalian cells [100] or Cho2p/Opi3p in yeast, likely also localizing to ER-mitochondria contacts [101], to yield phosphatidylcholine (PC).

Other lipids to be imported into mitochondria from the ER include phosphatidic acid (PA), the precursor for both ER-produced PS and mitochondrial cardiolipin [102]. While cardiolipin is essential for mitochondrial oxidative phosphorylation, apoptosis, mitochondrial protein import, mitophagy and mitochondrial membrane dynamics, surprisingly little is known about how its precursor PA reaches mitochondria from its origin on the ER in both mammalian and yeast model systems [103]. In yeast, cardiolipin synthesis from imported PA takes place within mitochondria, following PA intra-mitochondrial transport with Ups1p, as a cascade of lipid-modifying enzymes located on the IMM [104]. As a mechanistic link to mitochondrial membrane dynamics, PA promotes the production of the OMM protein Ugo1p, an activator of mitofusins [70]. Whether this mechanism operates in mammalian cells is highly doubtful, given that the recently characterized mammalian Ugo1-like protein SLC25A46 promotes mitochondrial fission [105]. Nevertheless, Ugo1p is probably just one example of how ER-mitochondria lipid metabolism can tie mitochondrial metabolism to mitochondria structure. Consistent with this hypothesis, PA inhibits the GTPase activity of dynamin-related protein 1 (Drp1) and, thus, blocks mitochondrial fission in mammalian cells [106].

### Interorganellar sterol transport at MCS: critical for lipid raft formation on the mammalian MAM and yeast MAM tethering

An additional group of molecules that may use MAMs as a conduit towards mitochondria are sterols [107]. Mammalian cholesterol and yeast ergosterol determine mitochondrial structure and function, despite not being very abundant within this organelle [108, 109]. However, there is evidence that the MAM might serve as a platform for sterol import in both mammalian and yeast cells. The mammalian steroidogenic acute regulatory (StAR) protein D1 (STARD1) shuttles free ER-derived cholesterol from the cytoplasm to the mitochondria in a PKA-dependent manner [110]. On the mitochondrial face of the MAM, sterols dock to the voltage-dependent anion channel (VDAC), which imports them to mitochondria [111] and distributes them within the organelle [112]. In yeast, Lam6p/Ltc1p, a StAR-domain containing protein localizes to MAMs [113], where it transfers sterols to mitochondria [114], but also increases ER-mitochondria tethering, via an interaction with the mitochondrial Tom70/71 complex [115]. Lam6 is not yeast-specific, but is conserved in mammalian models and increases the formation of MCS, not just between the ER and mitochondria, but also between MCS involving the nucleus and the endosomal system [115].

Aside from the shared function of sterol import into mitochondria at the MAM, the mammalian and *S. cerevisiae* model systems do not use sterols on the MAM structure in the same way. While several labs have demonstrated that the mammalian MAM is a cholesterol-rich lipid raft-like membrane [116–119] containing caveolin [120–122], yeast mitochondria lack raft structures and it is therefore unclear whether the yeast MAM exhibits raft properties [123]. Based on current information, this is another important distinction between yeast and mammalian MAM, since mammalian caveolin serves as an essential scaffold for enzymes mediating steroid and lipoprotein related processes at the MAM [124]. It is therefore no surprise that the removal of cholesterol from intracellular membranes upon addition of methyl β cyclodextrin disrupts the mammalian MAM [125], and impairs mitochondrial bioenergetics [126]. To our knowledge, the consequence for MAMs by this drug treatment has so far not been analyzed in yeast.

Inter-organellar sterol exchange is also mediated by the 16 mammalian oxysterol-binding protein (OSBP)-related proteins (ORPs) and 7 yeast oxysterol-homology (Osh) proteins, a class of proteins that frequently localize to MCS involving the ER [127]. Some, but not all ORP/Osh proteins target to the ER using a di-phenylalanine motif in an acidic stretch (FFAT) motif [128] and translocate sterols to a partner organelle by exchanging it for phosphatidylinositol 4-phosphate [129]. Within this class of proteins, mammalian ORP5 and ORP8 localize to ER-mitochondria contact sites, where they interact with a mitochondrial membrane protein, PTPIP51, and determine mitochondria membrane dynamics and function [130]. An open question is at the moment whether ORP/Osh proteins mediate sterol enrichment at the MAM itself.

### The mammalian MAM accommodates ER-mitochondria calcium signaling, a function not fully reproduced in the yeast model

While it is currently unknown whether the MAM provides a physical scaffold for proteins mediating mitochondrial oxidative phosphorylation [131], it does act as a transfer point for ER-derived Ca^2+^ needed for four mitochondrial dehydrogenases that serve as key stimulators of respiration and the Krebs cycle (Fig. 1) [132]. This connection explains why the MAM is necessary to maintain mitochondrial bioenergetics in mammalian cells [133]. While extracellular Ca^2+^ can also activate dehydrogenases, it is preferentially IP_3_R-released Ca^2+^ that has this function [134]. Ca^2+^ also controls the opening of the permeability transition pore, critical for mitochondrial protein content, as well as the mitochondrial membrane potential [135]. This connection had been discovered already in the early stages of research on ER-mitochondria contacts [136]. Interestingly, elevated cytosolic [Ca^2+^] also activates cholesterol import from the ER into mitochondria, as well as its intramitochondrial conversion [137, 138]. ER-derived Ca^2+^ also has a fundamental importance for the spatial positioning of mitochondria, since IP_3_R Ca^2+^ release arrests mitochondria movement [139]. The mechanistic basis for this observation is that under conditions of elevated cytosolic [Ca^2+^], the EF-hand Ca^2+^-binding GTPases MIRO1 and MIRO2 no longer facilitate the movement of mitochondria along microtubules [140–142]. In mammalian cells, this condition is triggered, for instance, upon ER stress and results in increased proximity between the ER and mitochondria [9, 63]. In this system, the activation of the unfolded protein response (UPR) coincides with increased ATP production and, thus, both mechanisms alleviate the accumulation of ER protein aggregates [143]. In yeast, the Ca^2+^-dependent mechanism cannot operate for a variety of reasons, but alteration of ER lipid composition, for instance by deletion of *OPI3*, is able to trigger the UPR as well [144], suggesting the formation of ER MCS, notably with mitochondria could be critical in this system as well.

Differences exist between mammalian and *S. cerevisiae* cells regarding mitochondrial Ca^2+^ handling. It was clear from the early 1970s that yeast mitochondria are unable to accumulate large amounts of Ca^2+^, such as the ones released from the ER [145], while mammalian cells were discovered in the 1980s to receive Ca^2+^ from the ER upon inositol 1,4,5-trisphosphate (IP_3_) exposure [146]. One reason for this difference in Ca^2+^ handling is that *S. cerevisiae* yeast stores most of its Ca^2+^ within vacuoles, which represent its lysosomal compartment [147], another is that their mitochondria lack the mitochondrial Ca^2+^ uniporter (MCU), a deficiency exploited for the experimental discovery of mammalian MCU [148–150]. Surprisingly, yeast mitochondria are still able to accumulate Ca^2+^ at low affinity, when provided with it in the growth medium, and this Ca^2+^ regulates mitochondrial dehydrogenases in yeast as well [151]. Potentially, this Ca^2+^ moiety enters *S. cerevisiae* mitochondria via an antiport activity that imports Ca^2+^ in exchange for two protons [152]. This mechanism is much less efficient than mammalian MCU and suggests that this yeast has simplified its Ca^2+^ machinery. Interestingly, other yeasts, such as *Endomyces magnusii*, have not undergone this simplification [153]. Therefore, while it has been suggested that yeast may serve as a model system for mitochondrial Ca^2+^ flux [154], it appears that this does not extend to MAM-specific flux or that models other than *S. cerevisiae* must be used that better align with mammalian cells.

It is therefore not surprising that the vast majority of knowledge regarding ER-mitochondria Ca^2+^ flux has been acquired from mammalian cell systems. Here, ER and mitochondria interact with each other to control the availability of cytosolic Ca^2+^ [155]. This interplay in turn determines the abundance of ER-mitochondria contacts, which decrease in the presence of EDTA [156]. The quasi-synaptic Ca^2+^ signal transmission between the ER and mitochondria [157] can be modulated and measured with artificial tethers containing Ca^2+^-detecting pericam [158]. Five to 20% of mitochondrial surface form contact sites with the ER [159], where Ca^2+^ microdomains allow mitochondrial MCUs to import Ca^2+^ [160] as the central part of a multisubunit protein complex comprising, among others, the gatekeepers Mitochondrial Calcium Uptake 1 and 2 (MICU1 and MICU2) [161–163].

An important function of this ion flux is to increase mitochondrial ATP production [164] and mitochondrial bioenergetics [165]. However, despite this important role, MCU ko cells are still able to produce ATP [166]. In contrast to mammalian cells, Ca^2+^ influx to mitochondria has unclear consequences for mitochondrial metabolism in *S. cerevisiae* yeast, with studies reporting both permeability transition pore opening [167] or closing [168], phenotypes potentially reflecting what happens in mammalian cells in a Ca^2+^-amount specific manner [135]. A currently poorly characterized link between the MAM and ATP has been described with studies on Sac1p in yeast [169]. This phosphatidylinositol 4-phosphatase allows ORP/Osh proteins to transport sterols by maintaining the phosphatidylinositol 4-phosphate gradient needed for this function [170, 171]. In yeast, Sac1p localizes to the ER [172]. Here, it is required for the import of mitochondrial ATP to the ER [169]. This connection between lipid metabolism and ATP import into the ER suggests Sac1p may fulfill a role for ER-mitochondria contacts, a likely location of import of mitochondrial ATP.

### Regulatory mechanisms of ER-mitochondria calcium signaling in metabolism and apoptosis in mammalian and yeast cells

The investigation of cytosolic Ca^2+^ waves in metazoan cell systems led to the discovery that ER chaperones are important regulators of ER-mitochondria Ca^2+^ signaling and, thus, connect ER protein folding to the formation and function of the MAM [173]. Ca^2+^ waves are excitatory events resulting from the ebb and flow of cytosolic [Ca^2+^] released via IP_3_Rs and taken up by the ER Ca^2+^ pump sarco/endoplasmic reticulum Ca^2+^-ATPase (SERCA) [174]. Ca^2+^ waves depend on mitochondria metabolism [175] and mitochondrial reactive oxygen species (ROS) [176]. These reactive molecules are released from mitochondrial cristae upon the arrival of [Ca^2+^] spikes at mitochondria, and subsequently accumulate on the MAM [177]. Here, they boost cytosolic Ca^2+^ oscillations via chemical sensitization of IP_3_Rs, thus creating a positive feedback loop [178]. Ca^2+^ waves therefore depend on the availability of Ca^2+^ on the cytosolic face of the ER. This amount is under the control of Ca^2+^ within the ER, and, hence, the activity of SERCA, as well as on the level of ER-mitochondria Ca^2+^ exchange that depends on the proximity of the two organelles.

ER chaperones such as calreticulin [179], calnexin [180] and ERp57 [181] inhibit cytosolic Ca^2+^ waves in a SERCA-dependent manner. By doing so, these chaperones determine the interplay between SERCA activity and ER-mitochondria proximity as well as mitochondrial metabolism. As one example, calnexin presumably activates SERCA, which results in increased Ca^2+^ accumulation within the ER and reduced Ca^2+^ transfer to mitochondria [182]. For this function, calnexin must localize to the MAM [182]. This enrichment to MAMs is under the control of Rab32 [183], a small GTPase that is not present in yeast [184]. This suggests that calnexin provides MAM-specificity to SERCA, which is instrumental in establishing normal ER-mitochondria Ca^2+^ communication, as shown by the compromising of this signaling upon inhibition of SERCA [185]. Opposing this function of calnexin is the MAM-localized oxidoreductase TMX1/TXNDC1, which inhibits SERCA2b in a thiol-specific manner and thus augments ER-mitochondria Ca^2+^ flux and mitochondrial metabolism [186]. A complementary mechanism is mediated by the ER oxidoreductase Ero1α, which activates IP_3_Rs at the MAM and also increases ER-mitochondria Ca^2+^ flux [187, 188].

As expected from the fundamental differences between mammalian and yeast ER-mitochondria Ca^2+^ handling, none of these mechanisms have been found faithfully reproduced in *S. cerevisiae*. Our own results show that *S. cerevisiae* calnexin and TMX1 (Eps1p) do not influence mitochondria metabolism, likely excluding a function for these ER proteins in yeast Ca^2+^ flux (J. Rockley and T. Simmen, unpublished results). Limited additional information is available from the fission yeast model system *Schizosaccharomyces pombe*. While mammalian MAM-localized calnexin allows  for proper ER Ca^2+^ filling [182] and normal apoptosis progression [189], *S. pombe* calnexin acts inhibitory to apoptosis pathways that stem from lipid imbalance due to inositol starvation [190]. Nevertheless, these findings identify calnexin as one of many connections between MAM chaperones and apoptosis.

These connections are based on massive Ca^2+^ diffusion towards mitochondria during the early phases apoptosis in mammalian cells [39]. The MAM determines the extent of apoptotic Ca^2+^ transfer from the ER to mitochondria, dependent on the binding of released cytochrome c to IP_3_Rs that get subsequently activated [191]. Likewise, crude cell death triggers such as hypotonic stress lead to a release of Ca^2+^ from the ER in *S. cerevisiae*, not unlike what happens in mammalian cells [192]. Despite the absence of an equivalent ER-mitochondria Ca^2+^ crosstalk in *S. cerevisiae*, yeast cells still show a rise of cytosolic Ca^2+^, accompanied by an initial acceleration of respiration upon the triggering of apoptosis [193]. In models with pheromone a factor that triggers apoptosis of haploid yeast in the absence of a mating partner [193] or with the antifungal amiodarone, yeast apoptosis depends on mitochondrial respiration [194]. It is not known at this point, whether yeast apoptosis requires Ca^2+^ diffusion towards mitochondria. However, yeast apoptosis involves the release of cytochrome c from mitochondria and a role for ROS [195]. Despite some parallels between the role of Ca^2+^ signaling in yeast and mammalian apoptosis, further research will have to determine whether and how yeast models reproduce the role of ER chaperones in apoptosis.

In mammalian cells, Bcl-2 family proteins also regulate MAM Ca^2+^ signaling [196]. For instance, ER-localized Bcl-x_L_ activates IP_3_R1 at low levels of [IP_3_] [197] to promote mitochondrial bioenergetics [198]. In contrast, the interaction between Bcl-2 and the IP_3_R largely serves to block pro-apoptotic Ca^2+^ transfer [199–202]. On the mitochondrial face of the MAM, Bcl-x_L_ also interacts with VDAC1 to inhibit apoptotic Ca^2+^ flux to mitochondria [203]. Whether the recently discovered *S. cerevisiae* Bcl-2 family protein [204] plays a role in ER-mitochondria Ca^2+^ flux as a death trigger like Bcl-2 family proteins in metazoan cells [205], is unknown at this point.

### Further MAM functions shared (or not) between mammalian and yeast cells

In addition to mitochondrial fusion, tied to MAM-localized ubiquitination of fusion proteins, the discovery of the BAP31-Fis1 protein complex at the MAM made one of the first described connections between proteins regulating mitochondrial membrane dynamics and the MAM [41]. This complex, also called the ARCosome, assembles under resting conditions, but acquires pro-caspase-8 upon apoptosis induction, which subsequently leads to BAP31 cleavage and Ca^2+^ transfer into mitochondria [41]. Yet another connection exists between mitochondrial fission  and the MAM. The GTPase Drp1 uses ER-mitochondria contacts as a basis for its oligomerization to subsequently mediate mitochondrial fission in both yeast and mammalian cells [15]. Another dynamin family member, dynamin-2, assists Drp1 in this task [206]. Interestingly, yeast lacking both Dnm1p and Fis1p reveal the tethering of mitochondria to cortical ER via the 313 kDa, pleckstrin-homology (PH)-domain containing protein Num1p [207], for which there is no obvious mammalian homologue. Unlike the association of Drp1/Dnm1p with the ER and mitochondria, the ARCosome has so far been demonstrated only in mammalian cells, despite the presence of BAP31 in yeast as well, where the BAP31-related Yet3p localizes close to the translocon [208] and the transcriptional repressor protein Opi1p [209].

Interestingly, the mammalian MAM is enriched for mitochondrial DNA (mtDNA)-protein complexes that associate with cholesterol [210]. Similarly, yeast mtDNA nucleoids associate with the ERMES complex [21]. The ERMES negative regulator Gem1p also acts to localize mtDNA [21], but it is currently unclear whether mammalian MIRO GTPases have the same function. The fact that C. elegans Miro mutations reduces the number of mtDNA suggests this might be the case [211].

A rather recent addition to the functional repertoire of the MAM is autophagy [16]. This degradative mechanism involves the formation of a double membrane that captures intracellular components to form an autophagosome and targets these to the lysosome [212]. While the MAM is now seen as prime source material for the formation of the autophagic isolation membrane, this is actually not far off from original suggestions from the late 1960s [213] and 1970s [214], when early studies in mammalian cells proposed the ER as the source of the isolation membrane. In yeast, this process takes place in a single location: the pre-autophagosomal site (PAS) [215] that is adjacent to the vacuole [216, 217], but it can form in several locations in mammalian cells [218]. Consistent with a role of the MAM in autophagy, lipid microdomains containing the ganglioside GD3 and the MAM marker calnexin form the autophagosomal membrane in the early autophagic process [219]. Moreover, using the MAM protein calnexin as a docking site upon hypoxia, the OMM protein FUNDC1 targets to the MAM to interact with Drp1 and mediate mitophagy [220]. This subtype of autophagy also depends on the transient interaction between mitochondrial and autophagosomal membranes in a mitofusin-2 dependent manner [221]. Subsequently, the MAM-localized mammalian t-SNARE Syntaxin-17 targets autophagosomes for fusion with endosomes and lysosomes [222]. The significance of syntaxin-17 for the MAM is further underscored by its localization to mitochondrial rafts and its requirement for Drp1 localization and activity at the MAM [223]. Moreover, syntaxin-17 targets the early autophagy marker Atg14 to MAM membranes [222], together with PACS-2 and mitofusin-2 [16]. Lastly, syntaxin-17 inhibits the PKA-anchoring protein (AKAP) Rab32. This small GTPase localizes to the MAM, where it determines chaperone enrichment to the MAM and activates Drp1 as an effector [184]. Together, these multiple connections between MAM proteins and autophagosome formation provide ample evidence and potential mechanisms that explain why MAM membranes have been identified as the point of origin for autophagosomes in mammalian cells.

Curiously, again showing that MAMs from yeast and mammalian cells differ, ERMES mutants do not have bulk autophagy defects, since they are able to recruit the autophagosome marker Atg8p to the vacuole normally, though they are impaired in the process of mitophagy [22]. The precise activity of ERMES appears to be to provide sufficient lipid supply from the ER for phagophore formation, following the formation of an Atg8p/Atg32p complex [224]. It is currently unclear whether the recently discovered yeast ER autophagic receptors Atg39p and Atg40p, homologs of mammalian FAM134B, play a role for MAM-initiated autophagy [225, 226]. While IP_3_R triple knockout cells show no apparent defect in ER-mitochondria apposition, they increase the number of their autophagosome formation [165], suggesting that Ca^2+^ flux is not needed for this MAM function in mammalian cells. In contrast, such a functional link exists for  mitofusin-2 [44] and PACS-2 [16]. Together, the intricacies of the autophagic function for mitochondria-ER contact sites require further research, in particular whether there are functional parallels between yeast and mammalian cells.

## Conclusion

The past decade has seen the advent of yeast *S. cerevisiae* as a model system to study the MAM. Research using this model system is powerful, but struggles with differences in the functioning of this MCS compared to mammalian cells. While these differences are not significant regarding lipid metabolism, differences of still unknown magnitude in ER-mitochondria Ca^2+^ flux currently preclude a 1:1 translation of findings between the two model systems (see Table 1). Further research on yeast apoptosis may reduce these in the future. Another deficiency is our lack of knowledge regarding yeast homologs of known mammalian tether proteins. Once these problems become resolved, we predict further rapid progress in the field using advantages of both systems to their fullest. This would then allow using the yeast model system for the further study of human disease, given the demonstrated role of the MAM and proteins regulating its function in neurodegeneration [227], cancer [228] and the metabolic syndrome [229, 230], to name but a few examples.

## Reviewers’ comments, round 1

### Reviewer’s report 1 Paola Pizzo, University of Padova

Reviewer comments:

The review by Herrera and Simmen analyses the central role of ER-mitochondria contact sites in different functionalities, with particular emphasis on human and yeast cells differences. It also describes some molecular complexes reported to be involved in ER-mitochondria tethering formation/modulation in the two systems. I have found the manuscript original, well-constructed and interesting for the research community. In particular, the part on lipid/sterol metabolism and transfer, as well as those on Ca2+ signaling and autophagy, is described in depth, leading to an efficient comparison between the two organisms. Perhaps, the authors should also spend some words on mitochondrial dynamics and mtDNA synthesis/distribution, two well established functions relying on ER-mitochondria connections that indeed present similarities, but also differences, in the two models. On the part describing the molecules involved in ER-mitochondria tethering, instead, I have some concerns. As also underlined by the authors, while in yeast the scenario is more defined, with two main protein complexes forming the tethering structures between the two organelles, in mammalian cells the situation is less clear, with a lot of proteins found to be localized at MAM, but with very few of them having tether characteristics. Among these, the authors deeply describes PACS-2, a cytosolic multifunctional sorting protein firstly reported to modulate ER-mitochondria juxtaposition. The protein, however, is involved in multiple cell pathways, making its direct role as a pure tether difficult to establish. As a general comment, I would suggest that, to date, it is difficult to define whether its reported role on ER-mitochondria tethering is direct or indirect. On the other hand, the molecular couple VAPB-PTPIP51, that they define “poorly characterized” (pg. 17), is, on my opinion, one of the best candidate for an ER-mitochondria tether structure since, upon genetic manipulation of each of the two proteins, modifications in both physical and functional (i.e., Ca2+ transfer) organelles coupling have been reported without any other evident alteration (De Vos KJ et al., Hum Mol Genet 2012; Stoica R et al., Nat Comm 2014). Likewise, the protein complex formed by IP3R, Grp75 and VDAC, reported by the authors as another tethering structure between ER and mitochondria in human cells, has to be considered, in my opinion, more a functional complex since DT40 cells knock-out for the three IP3R isoforms show, by EM analysis, unmodified ER-mitochondria physical association (Csordas et al., J Cell Biol 2006). The section on the role of mitofusin 2 as a tether is also not well discussed and misses several references not allowing the reader to have a complete picture of this debated issue. I am personally involved in this controversy, but I honestly think the cited literature is unbalanced. In fact, the tether role of this protein has been recently doubted by several independent groups, in different cell types and by different techniques (Cosson et al., PLoS One 2012; Filadi et al., PNAS 2015; Li et al., Mol Biol Cell 2015; Wang et al., J Cell Sci 2015; Leal et al., J Cell Mol Med 2016; Filadi et al., Cell Reports 2016). Thus, multiple biochemical, morphological, functional and genetic data actually demonstrate that Mfn2 acts as an ER-mitochondria tethering antagonist. For scientific clarity, I think the authors should be more comprehensive on this part and discuss better this critical point. For example, they report that “mitofusin-2 ko cells are resistant to apoptosis and show reduced Ca2+ transfer from the ER to mitochondria” quoting Munoz et al., 2013 (pg. 16); in this latter paper, however, no data on reduced Ca2+ transfer in Mfn2-KO cells, compared to wt, are presented; moreover, a defective apoptosis in Mfn2-KO cells was present only in response to specific ER stress-linked stimuli, such as tunicamycin or thapsigargin, and not upon stimulations with others apoptotic drugs acting with different mechanisms. These data were explained by the morphological observation that Mfn2 ablation causes abnormal ER expansion in response to ER stress. On the contrary, the review does not mention the finding that, upon acute Mfn2 down-regulation (to avoid any possible difference due to clonal adaptation), an higher cell death sensitivity has been reported (Filadi et al., PNAS 2015). Importantly, the latter result supports an increased ER-mitochondria Ca2+ transfer in Mfn2 silenced cells (thus, an increased ER-mitochondria vicinity) because it was only observed upon cell stimulation with ceramide (one of the most characterized agent inducing cell death linked to a mitochondrial Ca2+ overload toxicity). Likewise, the authors report: “Further demonstrating a role in the formation of ER-mitochondria contacts, mitofusin-2 ko cells show a clear decrease in mitochondrial respiration capacity”, quoting Mourier et al., 2015 (pg. 16). This elegant paper, however, demonstrates that the defect is due to reduced coenzyme Q levels and does not correlate it with ER-mitochondria contacts. Moreover, other findings are present in the literature: for example, the pivotal study of Chen et al. (2003) reportes a stochastic depolarization of individual mitochondria in Mfn2-KO cells, although the overall respiratory capacity of these cells was substantially unaffected (Chen et al., JCB 2003), and acute Mfn2 reduction has been shown not to change respiratory rate and ATP production, although a slightly reduced mitochondrial membrane potential (measured as TMRM fluorescence) was found (Filadi et al., PNAS 2015; Leal et al., J Cell Mol Med 2016). Finally, on the findings showing that in mouse MEFs (and not in human cells as erroneously reported) Mfn2 knock-out “apparently” leads to ER-mitochondria closer contacts (Cosson et al., PLoS One 2012; Filadi et al., PNAS 2015), the authors comment that: “since this effect has recently been shown to occur in a cell-culture dependent manner, these objections remain controversial and are likely due to a compensatory mechanism” (pg. 17), based on the recent paper by Scorrano’s group (Noan et al., PNAS 2016). The sentence is not adequate and must be revised. Pointing out that the increased contacts between ER and mitochondria in cells ablated of Mfn2 were measured (by quantitative EM) by both groups and not “apparent”, the paper by Naon et al. does not resolve at all the controversy on the role of Mfn2 in ER-mitochondria coupling (not addressing the relevant parameter, i.e., the number of contacts between the organelles, and not explaining the discrepancy between confocal and electron microscopy data on this aspect). In addition, decide which is the physiologically relevant cell density that validates the results appears, to say the least, trivial.

Minor Issues:

Several inconsistencies or imprecisions are present throughout the text. Some examples: 1. In the Summary the authors report: “ER-mitochondria contacts have originally been discovered in human cells…” but then in the Introduction they point out : “Endoplasmic reticulum (ER)-mitochondria contacts were described for the first time by Wilhelm Bernhard on electron micrographs of rat liver in 1952 [1] and 1956 [2, 3]”. The two sentences are contradictory. In general, the adjective “human” is sometimes used instead of “mammalian”. 2. The authors say that: “In liver and other human cells and cell lines, the distance between the ER and mitochondria is now recognized to typically measure 15–30 nm under resting conditions” (Introduction, lane 56). Actually, the thickness of the contacts between the two organelles can vary a lot, depending on which part of the ER mitochondria are connecting (see for example Giacomello and Pellegrini, Cell Death and Differentiation 2016, for a recent review on this issue). Thus, more than one type of contact exist between ER and mitochondria, likely linked to different functionalities. 3. In Table 1, on the specific function “MAM in apoptosis”, the authors report that in human cells “MAM Ca2+ transfer accelerates apoptosis” while in yeast “Cytoplasmic Ca2+ increase initially boosts mitochondrial respiration”. I think the statements are prone to being misunderstood, since also in mammals physiological cytoplasmic Ca2+ rises are linked to mitochondrial respiration and ATP production. Only an exaggerated ER-mitochondria Ca2+ transfer has been associated to a sensitization towards apoptotic stimuli (and a sub-threshold ER-mitochondria Ca2+ signal to autophagy). 4. Other disease-related molecules reported to be able to modulate ER-mitochondria tethering, such as presenilin 2, α-synuclein, DJ-1 and parkin are not even mentioned by the authors in the corresponding section.

Author’s response:

*1. We have added discussion on mitochondrial dynamics and the distribution of mtDNA. This is found as two new paragraphs on pages 21 and 22 (lines 456–477). These deal with mitochondrial fission and mtDNA. The section on mitofusin also discusses the role of ubiquitination on mitochondrial fusion.*

*2. We have specified that PACS-2 is “involved in MAM tethering”. We realize this cytosolic protein is unlikely a direct mediator of tethering. Nevertheless, it is functionally connected to BAP31, a known component of a tethering complex called the ARCosome. Since BAP31 undergoes cleavage in the absence of PACS-2, this connection provides a candidate mechanism how PACS-2 could functionally determine ER-mitochondria apposition. We also provide some limited information available about the potential role of Yet3p (yeast BAP31).*

*3. We have expanded discussion of the VAPB-PTPIP51 protein complex and include two novel studies describing regulatory proteins of this complex. We thank Dr. Pizzo for pointing out the result from DT40 cells that we have included in the revised manuscript to further characterize the IP3R-VDAC complex.*

*4. Regarding mitofusin-2, we have significantly altered the manuscript. We agree with Dr. Pizzo that several papers were not extensively discussed in the previous version. The reason for this omission was that the mitofusin-2 controversy is not at the center of this review. However, we agree with Dr. Pizzo that a better description of the issues at hand will be of interest for the readers of our yeast-mammalian system comparison, since it might impact what researchers might find about yeast Fzo1p.*

*We now state more clearly that a number of studies have found that “ER-mitochondria contacts increase upon mitofusin-2 depletion” (page 8, line 158, 159), thus making our discussion more comprehensive, as requested by Dr. Pizzo. We have also clarified the various observations on apoptosis induction as well as on respiration in Mfn2 depleted cells found by the research community. While we have absolutely no reason to doubt any published results on mitofusin-2, whether as a tether or inhibitor of tethering, we stand by the original statement that such inconsistencies could be based on cell culture conditions. In this sense, we are convinced that the discussion on mitofusin-2 is unnecessarily polarized, but could be reconciled as outlined in our revised manuscript. We now clearly label the identification of mitofusin-2 as a MAM tether as “our opinion”, but this statement is also based on our own expertise and experiments that we do not wish to revoke. But we do also provide additional findings for such an opinion: first, we now state that “we have found that mitofusin-2 ko cells lack fluorescence derived from an ER-mitochondria dimeric split green fluorescent protein” (Alford et al., 2012). Second, we now mention that mitofusin-2 depletion drastically alters intracellular calcium handling, especially at the level of the ER, which most studies do not account for. Third, we discuss that mitofusin-2 depletion is compensated by a variety of effects, not just downregulation of MCU, but most importantly the induction of ER stress, in absolute dependence on PERK (Munoz et al., 2013). The importance of this insight should not be underestimated and is critical for the understanding not just of mitofusin-2, but of the entire MAM! Since ER stress results in the increase of ER-mitochondria apposition, as reported by us (Bravo et al., 2011), and PERK is a tether itself, as reported by the Agostinis lab (Verfaillie et al., 2012), any study disputing a role of mitofusin-2 in tethering should investigate the presence of ER stress. This compensatory effect could single-handedly explain the entire controversy, again in our opinion. We conclude this section by stating that “Future studies involving more detailed characterizations of intracellular Ca2+ handling and the ER phenotype will have to untangle the equally important roles of mitofusin-2 in ER-mitochondria tethering and in the prevention of ER stress”. Together, we hope that while not the focus of this review, the revised section more accurately reflects the current information on this controversial topic and provides our interpretation of the results available today, clearly labeled as “our opinion”.*

*5. We have corrected the usage of the word “human” to “mammalian” to simplify and correct our text.*

*6. We further describe the thickness of the MAM as 15–30 nm “in contact sites” and mention that it is not fully understood how it can become tighter.*

*7. We have revised the table as requested.*

*8. We have added a couple of review articles to discuss the role of the MAM in disease. However, this was limited to one sentence, as a more in depth discussion of the MAM in disease is outside the scope of this article.*

### Reviewer’s report 2 Maya Schuldiner, Weizmann Institute of Science (nominated by Luca Pellegrini, University of Laval)

Reviewer comments:

To promote maximal clarity and usefulness for readers we can suggest a couple of small points: 1.) The title “Of yeast and men: MAMs and MERCs come in two flavors” is a bit misleading, as it gives the impression that MAMs and MERCs are different things or that MAMs are in yeast and MERCs in humans (which is not the case). Throughout the text the two names are also often interchanged and this also adds confusion. We recommend to just pick one name for the title and throughout the text (just mentioning the other name once). 2.) At the mechanistic level, some MAM functions are not easy to grasp for readers that are non specialists in the field. Accessibility to a broad readership would very much benefit from illustration of these processes by figures. We suggest to include figures at least on the following topics: a.) Overview over the MAM contact site machineries in mammals and in yeast b.) MAMs and lipid metabolism C.) Calcium signaling at MAMs 3.) At the current position, the chapter “Proteins mediating formation of the MAM” is sandwiched between two chapters on function. We suggest to place it directly after the introduction and then have the rest of the manuscript describe functions of MAMs. Moving the chapter will only require minor alterations to the text. Outlining the protein machineries involved in MAMs early in the manuscript will also improve accessibility to readers unfamiliar with the topic. 4.) MAMs have an intriguing role in mitochondrial fission and nucleoid positioning, which is currently only mentioned in one sentence. We suggest to include a full chapter on this important MAM function. 5.) The chapter “Further MAM functions shared (or not) between human and yeast cells” only deals with roles of MAMs in autophagy and mitophagy. We suggest to change the title accordingly. 6.) Regarding the same chapter, the authors should include a paragraph on the beautiful work from the Benedikt Westermann lab on the role of ERMES mediated MAMs in mitophagy. 7.) Minor points: a.) The comparison between mammalian and yeast MAMs is the main focus of the review. Maybe it would be helpful to divide each chapter in three parts (mammalian; yeast; comparison)? b.) Sometimes technical terms, abbreviations and names are not introduced or not sufficiently explained. Examples: L142: Drp1 L167: methyl beta cyclodextrin L241: ROS L269: S. pombe L278: pheromone a factor L279: amiodarone L343: ko cell c.) L26-28: “Like other MCS, ER-mitochondria contacts have originally been discovered in human cells, where they have been designated as mitochondria-associated membranes (MAMs)”. Since numerous contacts were discovered first in yeast, we suggest to omit the “like other MCS”. d.) L33: The authors might consider mentioning the MAM components that are conserved in mammals (Lam6, Gem1) in addition to ERMES. e.) L57: “(…) the distance between the ER and mitochondria is now recognized to typically measure 15–30 nm under resting conditions”. For clarity consider including “in contact sites”. f.) L98: “Whether all of these enzymes use mitochondrial targeting sequences to localize to the MAM (…)”. The term mitochondrial targeting sequence is generally used for proteins with a cleavable N-terminal signal directing them to the mitochondrial matrix or inner membrane. We suggest to write instead “it is currently unclear how these enzymes are targeted…” g.) L173: To our knowledge, not all ORP/Osh proteins contain FFAT motifs, at least in yeast this is the case. h.) L182: “In addition to providing the physical scaffold for mitochondrial oxidative phosphorylation, the MAM also serves (…)” Could the authors explain this statement in more detail and provide references as MAMs are not usually considered necessary for ox-phos processes? i.) L193 onwards: “…The reason for this difference in Ca2+ handling is that S. cerevisiae yeast mitochondria lack the mitochondrial Ca2+ uniporter (MCU)”. Another important difference is the fact that in yeast, the ER is not the main place of calcium storage in the cell but rather the vacuole (yeast lysosome). The authors might want to include this into the text. j.) P11, first paragraph: In the section on MAMs role in calcium we recommend to include a few sentences on Gem1, which is a calcium binding MAM component conserved from yeast to human. k.) L301-302: “The S. cerevisiae model currently shows an advantage due to its screening power that has led to the identification of two tethering complexes so far.” Numerous tethering components have been suggested also in mammals, so this statement could be toned down. l.) L314: The authors might consider to cite van der Laan et al., 2012, Trends Cell Biol, who first coined the term ERMIONE. m.) L322: Please mention Gem1, which is conserved in mammals. n.) L325: It is currently unclear whether the EMC acts as the PS transfer machinery or simply affects PS transfer so this statement should be altered. o.) L328: “EMC appears to moonlight as a chaperone”. It is currently unclear what is the primary function of EMC. We recommend to tone this statement down. p.) L342: Yeast also has a Fis1 homolog which the authors may want to mention. q.) L353-354: FZO1 is a key component in mitochondrial fusion, and this function alone is sufficient to explain the growth phenotypes. Therefore, these phenotypes do not automatically suggest that Fzo1 acts as a tether.

Author’s response:

*1. We have revised the title as requested and have reduced the usage of MERCs to a few instances, using most of the time the more familiar MAM. We left the MERC term in the abstract, as it is currently unclear whether this acronym will gain more traction in the future.*

*2. We have added two figures, as requested by Dr. Schuldiner that summarize the known players and mechanisms in ER-mitochondria tethering, lipid metabolism and calcium signaling.*

*3. We have moved the section on MAM tethers to right after the introduction, as requested. This has necessitated the introduction of a few transition sentences to accommodate it better at its new position.*

*4. As also requested by Dr. Pizzo, we now discuss mitochondrial fission on page 21, lines 456–471. This had led us to keep the title of this last chapter as is, since we now have added two more functions (fission and distribution of mtDNA).*

*5. We have added discussion of Dr. Westermann’s work, since we agree 100% that this is essential work: “ERMES mutants do not have bulk autophagy defects, since they are able to recruit the autophagosome marker Atg8p to the vacuole normally, though they are impaired in the process of mitophagy (Bockler and Westermann, 2014a). The precise activity of ERMES appears to be to provide sufficient lipid supply from the ER for phagophore formation, following the formation of an Atg8p/Atg32p complex (Bockler and Westermann, 2014b)”.*

*6. We have corrected the introduction of acronyms and have provided description of pheromone a factor/amiodarone-mediated apoptosis.*

*7. We state in several spots the critical observation by Dr. Schuldiner that Gem1 and Lam6 are conserved, notably in the abstract: “This has led to the discovery of novel MAM tethers such as the yeast ER-mitochondria encounter structure (ERMES), absent in the mammalian system, but whose regulators Gem1 and Lam6 are conserved.” It will exciting to see how these two proteins influence ER-mitochondria contacts (and other MCS) in the mammalian system.*

*8. We have refined the description of FFAT motifs for ORP/Osh proteins.*

*9. We have refined the introduction of the MAM for intramitochondrial localization: “While it is currently unknown whether the MAM provides a physical scaffold for proteins mediating mitochondrial oxidative phosphorylation (Gellerich et al., 2010), it does act as a transfer point for ER-derived Ca2+ needed for four mitochondrial dehydrogenases that serve as key stimulators of respiration and the Krebs cycle (Fig.* 1 *) (Denton, 2009).”*

*10. We thank Dr. Schuldiner for reminding us about the role of the vacuole for yeast calcium handling. This was inadvertently cut from the previous version. It is now found on page 17, lines 351, 352.*

*11. We have modified the sentence about the power of yeast as a model system as follows (page 5/6): “While the S. cerevisiae model has taken advantage of genetic screening power that has led to the identification of two tethering complexes so far, mammalian systems currently benefit from a larger array of functional readouts of the contacts (a summary of the main proteins involved in ER-mitochondria tethering in yeast and mammalian cells is shown in Fig.* 1 *).”*

*12. We now cite the van der Laan paper (new reference 24).*

*13. We have rephrased the description of EMC for PS trafficking as follows (line 124, 125): “Yeast EMC plays a role for the import of PS into mitochondria, but this could be direct or indirect”. We have also altered the statement regarding EMC moonlighting as a chaperone (lines 128–131): “Here, in addition to tethering mitochondria to the ER, EMC also acts as a chaperone for the assembly of multipass transmembrane proteins (Satoh et al., 2015). Further research will have to determine which of these functions is the main role of EMC.”*

*14. We have added that Fis1 is also found in yeast (line 145/146).*

*15. We have modified the discussion about Fzo1 (lines 184–187): “Yeast deleted of FZO1 do not grow well on fermentable carbon sources and exhibit a petite phenotype, which would be expected if it were a tether; alternatively, this property could depend solely on its role in mitochondrial fusion, two hypotheses to be tested in the future.”*

### Reviewer’s report 3 György Szabadkai, University College London

Reviewer comments:

1. The metabolic pathways involving PA, PS, PE and cardiolipin cycling and synthesis between the ER and mitochondria and the participating enzymes should be described in a short paragraph, or summarized in a small scheme. 2. Steroid synthesis in mammalian cells occurs by interactions between the ER and mitochondria, which is regulated by ER-mitochondrial Ca2+ transfer. Although no specific transporters of the steroid intermediates have been described, the process would need a mention in the sterol chapter. (see e.g., https://www.ncbi.nlm.nih.gov/pubmed/15044681) 3. A general account for the role of stress responses – linked to both adaptive responses (see e.g., https://www.ncbi.nlm.nih.gov/pubmed/21628424) and apoptotic responses is missing. The ER stress response is evolutionarily well conserved and seems to strongly regulate ER-mitochondrial interactions in mammals, thus one would expect a similar process being in place in yeast?

Author’s Response:

*1. We have increased discussion of PS/PE metabolism on page 12 (lines 259–262) and we have also added the new Fig.* 2 *. Both were very useful suggestions that have improved the manuscript.*

*2. We provide a brief discussion of the role of calcium in sterol synthesis and trafficking on page 15 (line 332. 333).*

*3. Following this, we also discuss the role of stress responses and their connection to lipids (lines 338–345). We felt that both points 2 and 3 fit better into the calcium chapter, since the reader can fit them better into the general scheme at this point.*

## Reviewers’ comments round 2

### Reviewer’s report 1 Paola Pizzo, University of Padova

Reviewer comments:

The authors have addressed all my concerns and the revised manuscript is now, in my opinion, more complete. In particular, I have appreciated their efforts in facing the debated role of Mfn2 in ER-mitochondria tethering, suggesting the possible role played by ER stress (associated to Mfn2 depletion) in determining organelles phenotype and their consequent relationship (features, so far, not sufficiently investigated). I agree that this contribution is not focused on the Mfn2 controversy, but since the protein is extensively reported to represent the main mammalian tether between the two organelles, I really think the issue deserves a critical evaluation. For this reason, I still have some notes on what the authors reported in the manuscript: 1. Page 8, lane 157: the authors do note quote all the papers that, so far, showed a negative role for Mfn2 in ER-mitochondria tethering, referring only the two original papers that firstly performed a quantitative EM analysis in wt and Mfn2 KO/KD MEFs and reported an increased number of ER-mitochondria contacts in Mfn2 ablated/depleted cells. As I said in my previous comments, the tether role of this protein has been, instead, doubted by several independent groups, in different cell types and by different techniques (Li et al., Mol Biol Cell 2015; Wang et al., J Cell Sci 2015; Leal et al., J Cell Mol Med 2016; Filadi et al., Cell Reports 2016). In particular, the paper by Wang et al. (J Cell Sci 2015), showing by EM that the knock-down of Mfn2 increases RER–mitochondria contacts in HT-1080 fibrosarcoma cancer cells, should be also cited (it is cited later), instead of Naon et al. (PNAS 2016), here erroneously quoted. 2. Page 9, lane 168: the explanation that Mfn2 KO/KD cells show an increased mitochondrial Ca2+ uptake, upon cell stimulation, because they present a higher ER Ca2+ content is not correct. In particular, the authors report erroneously our results (Filadi et al., PNAS 2015): we have shown that in Mfn2 KD MEFs (acutely down-regulated by specific siRNAs), where no variation in MCU expression occurs, an increased mitochondrial Ca2+ uptake, compared to controls, is revealed even upon similar IP3-induced cytosolic Ca2+ rises. 3. Page 9, lane 171: the authors explain the published result of MCU down-regulation in Mfn2 KO cells (Filadi et al., PNAS 2015), suggesting a compensatory mechanism that happens in cell culture-manner, as reported by Naon et al. (PNAS 2016). It must be noted, however that a downregulation of MCU expression in cell culture-manner has been reported only for wt and not for Mfn2 KO cells (Naon et al., PNAS 2016), thus not explaining the original observation.

Author’s Response:

*1. In agreement with Dr. Pizzo, we have expanded the discussion of papers investigating mitofusins and the MAM, previously kept limited to not distract from the main topic of this review. This approach is also reflected in our new statement about the controversy, since we believe that* ***“these observations are likely only apparently contradictory and they could be based on incomplete characterizations of mitofusin-2 ko or knockdown cells in some studies.”*** *i. We have added the studies on presenilin-2. These studies show that presenilin-2 over-expression, in particular of AD-linked mutant presenilin-2, leads to a shift of Ca* ^*2+*^ *content from the ER to mitochondria, accompanied by accelerated flux of Ca* ^*2+*^ *from the ER due to reduced SERCA activity and increased leak. These observations are 100% identical to what we have observed with TMX1 over-expression, another modulator of SERCA activity. Regarding the significance of the combinatory interventions on presenilin-2 and mitofusin-2, we now state in the text that the* ***“consequence of mitofusin-2 depletion” to boost MAMs “is not further increased in the presence of presenilin-2 mutant protein”*** *. Regarding the role of presenilin-2 for ER-mitochondria tethering and Ca* ^*2+*^ *handling, we now state that this activator of SERCA* ***“normally increases ER-mitochondria tethering in wild type cells due to a relative shift of Ca*** ^***2+***^ ***content from the ER to mitochondria.”*** *ii. We also decided to discuss the papers from the Nabi lab, but this discussion was separated from the other papers, not least due to the complexity of their findings, but also for the following reasons: The first paper shows that Gp78 preferentially degrades mitofusin-1 (Figure 7B, (Li et al., 2015), while the second paper shows that knockdown of mitofusin-1 increases sER-mitochondria contacts, but no effect is seen for single mitofusin-2 knockdown (Figure 4B, (Wang et al., 2015). The synthesis of the results by the two papers suggests that under the experimental conditions used by the Nabi lab, mitofusin-2 knockdown had no effect on MAM formation by itself (Figure 4A, Wang et al., 2015), and only rescued the reduced formation of MAM formed from rER (Figure 4B, Wang et al., 2015) upon Gp78 knockdown. Together, this indicates that the papers from the Nabi lab do not provide evidence for either mitofusin-2 hypothesis, maybe due to incomplete knockdown, but rather clearly suggest mitofusins play a role in the ratio of rER/sER-mitochondria contacts. We therefore state that the* ***“mitofusins act in concert to reduce MAM overall, and that the respective expression levels of mitofusins determines the ratio of sER/rER-mitochondria contact formation. However, in our opinion, they do not shift the balance significantly towards either hypothesis on the role of mitofusin-2 for MAMs, since mitofusin-2 knockdown alone had no measurable effect on MAMs in this system.”***

*2. We recognize and apologize for the lack of clarity regarding the effects seen for cytosolic Ca* ^*2+*^ *, especially from this reviewer’s papers. We now state that altered* ***“global cellular Ca*** ^***2+***^ ***handling at the ER-mitochondria interface (Bravo et al., 2011, Bravo-Sagua et al., 2016, Csordas et al., 2006)”*** *… upon ER stress could complicate interpretation of MAM Ca2+ handling, …* ***“even when the cytosolic response is kept even (Filadi et al., 2015).”***

*3. Addressing the second comment without confusing the reader (who is not looking for a profound discussion of the differences of individual papers on mitofusin-2) is not easy. We now state that the* ***“downregulation of the MCU,”*** *…is* ***…“observed to varying extents (Naon et al., 2016) in Mfn2−/− cells (Filadi et al., 2015), but not in cells, where mitofusin-2 has been knocked down (Filadi et al., 2015)”.***

References for Reviewers’ Comments Rounds 1 and 2

Bravo R, Vicencio JM, Parra V, Troncoso R, Munoz JP, Bui M, Quiroga C, Rodriguez AE, Verdejo HE, Ferreira J, Iglewski M, Chiong M, Simmen T, Zorzano A, Hill JA, Rothermel BA, Szabadkai G, Lavandero S (2011) Increased ER-mitochondrial coupling promotes mitochondrial respiration and bioenergetics during early phases of ER stress. J Cell Sci 124: 2143–52

Bravo-Sagua R, Lopez-Crisosto C, Parra V, Rodriguez-Pena M, Rothermel BA, Quest AF, Lavandero S (2016) mTORC1 inhibitor rapamycin and ER stressor tunicamycin induce differential patterns of ER-mitochondria coupling. Sci Rep 6: 36394

Csordas G, Renken C, Varnai P, Walter L, Weaver D, Buttle KF, Balla T, Mannella CA, Hajnoczky G (2006) Structural and functional features and significance of the physical linkage between ER and mitochondria. J Cell Biol 174: 915–21

Filadi R, Greotti E, Turacchio G, Luini A, Pozzan T, Pizzo P (2015) Mitofusin 2 ablation increases endoplasmic reticulum-mitochondria coupling. Proc Natl Acad Sci U S A 112: E2174-81

Li L, Gao G, Shankar J, Joshi B, Foster LJ, Nabi IR (2015) p38 MAP kinase-dependent phosphorylation of the Gp78 E3 ubiquitin ligase controls ER-mitochondria association and mitochondria motility. Mol Biol Cell 26: 3828–40

Naon D, Zaninello M, Giacomello M, Varanita T, Grespi F, Lakshminaranayan S, Serafini A, Semenzato M, Herkenne S, Hernandez-Alvarez MI, Zorzano A, De Stefani D, Dorn GW, 2nd, Scorrano L (2016) Critical reappraisal confirms that Mitofusin 2 is an endoplasmic reticulum-mitochondria tether. Proc Natl Acad Sci U S A

Wang PT, Garcin PO, Fu M, Masoudi M, St-Pierre P, Pante N, Nabi IR (2015) Distinct mechanisms controlling rough and smooth endoplasmic reticulum contacts with mitochondria. J Cell Sci 128: 2759–65

## Abbreviations

AKAP PKA: Anchoring protein
ATP: Adenosine trisphosphate
BAP31: B cell receptor associated protein of 31 kilodalton
BHK: Baby hamster kidney
Drp1: Dynamin related protein 1
EMC: ER membrane protein complex
ER: Endoplasmic reticulum
ERMES: ER-mitochondria encounter structure
ERMIONE: ER-mitochondria organizing network
FAM134B: Family with sequence similarity 134 member B
FFAT: Di-phenylalanine motif in an acidic stretch
FUNDC1: FUN14 domain containing protein 1
FUS: Fused in sarcoma
GTPase: Enzyme hydrolyzing guanosine triphosphate
IMM: Inner mitochondrial membrane
ko: Knockout
MAM: Mitochondria-associated membrane
MCS: Membrane contact site
MCU: Mitochondrial calcium uniporter
MERCs: Mitochondria-ER contacts
Mfn2: Mitofusin-2
MICOS: Mitochondrial contact site complex
MICU: Mitochondrial calcium uptake
MIRO: Mitochondrial Rho GTPase
MITOL: Mitochondrial ubiquitin ligase
mtDNA: Mitochondrial DNA
OMM: Outer mitochondrial membrane
ORP: Oxysterol-binding protein (OSBP)-related proteins
OSBP: Oxysterol-binding protein
PA: Phosphatidic acid
PACS-2: Phosphofurin acidic cluster sorting protein 2
PAS: Preautophagosomal site
PC: Phosphatidylcholine
PE: Phosphatidylethanolamine
PEMT: Phosphatidylethanolamine methyltransferase
PERK: Protein kinase R (PKR)-like endoplasmic reticulum kinase
PH: Pleckstrin homology
PS: Phosphatidylserine
PTPIP51: Protein tyrosine phosphatase-interacting protein 51
SAM: Sorting and assembly machinery
SERCA: Sarco/endoplasmic reticulum Ca^2+^-ATPase
StAR: Steroidogenic acute regulatory
STARD1: Steroidogenic acute regulatory (StAR) protein D1
TMX1/TXNDC1: Thioredoxin containing membrane protein 1
UPR: Unfolded protein response
VAPB: Vesicle-associated membrane protein- associated protein B
VDAC: Voltage-dependent anion channel

## References

[1] Bernhard W, Haguenau F, Gautier A, Oberling C (1952). [Submicroscopical structure of cytoplasmic basophils in the liver, pancreas and salivary gland; study of ultrafine slices by electron microscope]. Z Zellforsch Mikrosk Anat.

[2] Bernhard W, Rouiller C (1956). Close topographical relationship between mitochondria and ergastoplasm of liver cells in a definite phase of cellular activity. J Biophys Biochem Cytol.

[3] Bernhard W, Rouiller C (1956). Microbodies and the problem of mitochondrial regeneration in liver cells. J Biophys Biochem Cytol.

[4] Vance JE (1990). Phospholipid synthesis in a membrane fraction associated with mitochondria. J Biol Chem.

[5] Rusinol AE, Cui Z, Chen MH, Vance JE (1994). A unique mitochondria-associated membrane fraction from rat liver has a high capacity for lipid synthesis and contains pre-Golgi secretory proteins including nascent lipoproteins. J Biol Chem.

[6] Sood A, Jeyaraju DV, Prudent J, Caron A, Lemieux P, McBride HM, Laplante M, Toth K, Pellegrini L (2014). A Mitofusin-2-dependent inactivating cleavage of Opa1 links changes in mitochondria cristae and ER contacts in the postprandial liver. Proc Natl Acad Sci U S A.

[7] Giacomello M, Pellegrini L (2016). The coming of age of the mitochondria-ER contact: a matter of thickness. Cell Death Differ.

[8] Vance JE (2015). Phospholipid synthesis and transport in mammalian cells. Traffic.

[9] Csordas G, Renken C, Varnai P, Walter L, Weaver D, Buttle KF, Balla T, Mannella CA, Hajnoczky G (2006). Structural and functional features and significance of the physical linkage between ER and mitochondria. J Cell Biol.

[10] Riezman H, Hay R, Gasser S, Daum G, Schneider G, Witte C, Schatz G (1983). The outer membrane of yeast mitochondria: isolation of outside-out sealed vesicles. EMBO J.

[11] Schatz G, Tuppy H, Klima J (1963). Trennung und Charakterisierung cytoplasmatischer Partikel aus normaler und atmungsdefekter Baeckerhefe. Z Naturforschung.

[12] Damsky CH (1976). Environmentally induced changes in mitochondria and endoplasmic reticulum of Saccharomyces carlsbergensis yeast. J Cell Biol.

[13] Elbaz Y, Schuldiner M (2011). Staying in touch: the molecular era of organelle contact sites. Trends Biochem Sci.

[14] Levine TP, Patel S (2016). Signalling at membrane contact sites: two membranes come together to handle second messengers. Curr Opin Cell Biol.

[15] Friedman JR, Lackner LL, West M, DiBenedetto JR, Nunnari J, Voeltz GK (2011). ER tubules mark sites of mitochondrial division. Science.

[16] Hamasaki M, Furuta N, Matsuda A, Nezu A, Yamamoto A, Fujita N, Oomori H, Noda T, Haraguchi T, Hiraoka Y (2013). Autophagosomes form at ER-mitochondria contact sites. Nature.

[17] Achleitner G, Gaigg B, Krasser A, Kainersdorfer E, Kohlwein SD, Perktold A, Zellnig G, Daum G (1999). Association between the endoplasmic reticulum and mitochondria of yeast facilitates interorganelle transport of phospholipids through membrane contact. Eur J Biochem.

[18] Kornmann B, Currie E, Collins SR, Schuldiner M, Nunnari J, Weissman JS, Walter P (2009). An ER-mitochondria tethering complex revealed by a synthetic biology screen. Science.

[19] Lang A, John Peter AT, Kornmann B (2015). ER-mitochondria contact sites in yeast: beyond the myths of ERMES. Curr Opin Cell Biol.

[20] Kornmann B, Osman C, Walter P (2011). The conserved GTPase Gem1 regulates endoplasmic reticulum-mitochondria connections. Proc Natl Acad Sci U S A.

[21] Murley A, Lackner LL, Osman C, West M, Voeltz GK, Walter P, Nunnari J (2013). ER-associated mitochondrial division links the distribution of mitochondria and mitochondrial DNA in yeast. Elife.

[22] Bockler S, Westermann B (2014). ER-mitochondria contacts as sites of mitophagosome formation. Autophagy.

[23] Wideman JG, Munoz-Gomez SA (2016). The evolution of ERMIONE in mitochondrial biogenesis and lipid homeostasis: an evolutionary view from comparative cell biology. Biochim Biophys Acta.

[24] van der Laan M, Bohnert M, Wiedemann N, Pfanner N (2012). Role of MINOS in mitochondrial membrane architecture and biogenesis. Trends Cell Biol.

[25] Ardail D, Gasnier F, Lerme F, Simonot C, Louisot P, Gateau-Roesch O (1993). Involvement of mitochondrial contact sites in the subcellular compartmentalization of phospholipid biosynthetic enzymes. J Biol Chem.

[26] Ardail D, Lerme F, Louisot P (1991). Involvement of contact sites in phosphatidylserine import into liver mitochondria. J Biol Chem.

[27] Hackenbrock CR (1968). Chemical and physical fixation of isolated mitochondria in low-energy and high-energy states. Proc Natl Acad Sci U S A.

[28] van der Laan M, Horvath SE, Pfanner N (2016). Mitochondrial contact site and cristae organizing system. Curr Opin Cell Biol.

[29] Aaltonen MJ, Friedman JR, Osman C, Salin B, di Rago JP, Nunnari J, Langer T, Tatsuta T (2016). MICOS and phospholipid transfer by Ups2-Mdm35 organize membrane lipid synthesis in mitochondria. J Cell Biol.

[30] Wideman JG, Gawryluk RM, Gray MW, Dacks JB (2013). The ancient and widespread nature of the ER-mitochondria encounter structure. Mol Biol Evol.

[31] Guarani V, McNeill EM, Paulo JA, Huttlin EL, Frohlich F, Gygi SP, Van Vactor D, Harper JW. QIL1 is a novel mitochondrial protein required for MICOS complex stability and cristae morphology. Elife 2015;4. 10.7554/eLife.06265 PMC4439739 25997101

[32] Ott C, Dorsch E, Fraunholz M, Straub S, Kozjak-Pavlovic V (2015). Detailed analysis of the human mitochondrial contact site complex indicate a hierarchy of subunits. PLoS One.

[33] Xie J, Marusich MF, Souda P, Whitelegge J, Capaldi RA (2007). The mitochondrial inner membrane protein mitofilin exists as a complex with SAM50, metaxins 1 and 2, coiled-coil-helix coiled-coil-helix domain-containing protein 3 and 6 and DnaJC11. FEBS Lett.

[34] Lahiri S, Chao JT, Tavassoli S, Wong AK, Choudhary V, Young BP, Loewen CJ, Prinz WA (2014). A conserved endoplasmic reticulum membrane protein complex (EMC) facilitates phospholipid transfer from the ER to mitochondria. PLoS Biol.

[35] Wideman JG (2015). The ubiquitous and ancient ER membrane protein complex (EMC): tether or not?. F1000Res.

[36] Satoh T, Ohba A, Liu Z, Inagaki T, Satoh AK. dPob/EMC is essential for biosynthesis of rhodopsin and other multi-pass membrane proteins in Drosophila photoreceptors. Elife. 2015;4. 10.7554/eLife.06306 PMC4341237 25715730

[37] Filippin L, Magalhaes PJ, Di Benedetto G, Colella M, Pozzan T (2003). Stable interactions between mitochondria and endoplasmic reticulum allow rapid accumulation of calcium in a subpopulation of mitochondria. J Biol Chem.

[38] Simmen T, Aslan JE, Blagoveshchenskaya AD, Thomas L, Wan L, Xiang Y, Feliciangeli SF, Hung CH, Crump CM, Thomas G (2005). PACS-2 controls endoplasmic reticulum-mitochondria communication and Bid-mediated apoptosis. Embo J.

[39] Baffy G, Miyashita T, Williamson JR, Reed JC (1993). Apoptosis induced by withdrawal of interleukin-3 (IL-3) from an IL-3-dependent hematopoietic cell line is associated with repartitioning of intracellular calcium and is blocked by enforced Bcl-2 oncoprotein production. J Biol Chem.

[40] Betz C, Stracka D, Prescianotto-Baschong C, Frieden M, Demaurex N, Hall MN (2013). Feature Article: mTOR complex 2-Akt signaling at mitochondria-associated endoplasmic reticulum membranes (MAM) regulates mitochondrial physiology. Proc Natl Acad Sci U S A.

[41] Iwasawa R, Mahul-Mellier AL, Datler C, Pazarentzos E, Grimm S (2011). Fis1 and Bap31 bridge the mitochondria-ER interface to establish a platform for apoptosis induction. EMBO J.

[42] de Brito OM, Scorrano L (2008). Mitofusin 2 tethers endoplasmic reticulum to mitochondria. Nature.

[43] Alford SC, Ding Y, Simmen T, Campbell RE (2012). Dimerization-dependent green and yellow fluorescent proteins. ACS Synth Biol.

[44] Munoz JP, Ivanova S, Sanchez-Wandelmer J, Martinez-Cristobal P, Noguera E, Sancho A, Diaz-Ramos A, Hernandez-Alvarez MI, Sebastian D, Mauvezin C (2013). Mfn2 modulates the UPR and mitochondrial function via repression of PERK. EMBO J.

[45] Papanicolaou KN, Khairallah RJ, Ngoh GA, Chikando A, Luptak I, O’Shea KM, Riley DD, Lugus JJ, Colucci WS, Lederer WJ (2011). Mitofusin-2 maintains mitochondrial structure and contributes to stress-induced permeability transition in cardiac myocytes. Mol Cell Biol.

[46] Guo X, Chen KH, Guo Y, Liao H, Tang J, Xiao RP (2007). Mitofusin 2 triggers vascular smooth muscle cell apoptosis via mitochondrial death pathway. Circ Res.

[47] Wan-Xin T, Tian-Lei C, Ben W, Wei-Hua W, Ping F (2012). Effect of mitofusin 2 overexpression on the proliferation and apoptosis of high-glucose-induced rat glomerular mesangial cells. J Nephrol.

[48] Wang W, Xie Q, Zhou X, Yao J, Zhu X, Huang P, Zhang L, Wei J, Xie H, Zhou L (2015). Mitofusin-2 triggers mitochondria Ca2+ influx from the endoplasmic reticulum to induce apoptosis in hepatocellular carcinoma cells. Cancer Lett.

[49] Sebastian D, Hernandez-Alvarez MI, Segales J, Sorianello E, Munoz JP, Sala D, Waget A, Liesa M, Paz JC, Gopalacharyulu P (2012). Mitofusin 2 (Mfn2) links mitochondrial and endoplasmic reticulum function with insulin signaling and is essential for normal glucose homeostasis. Proc Natl Acad Sci U S A.

[50] Cosson P, Marchetti A, Ravazzola M, Orci L (2012). Mitofusin-2 independent juxtaposition of endoplasmic reticulum and mitochondria: an ultrastructural study. PLoS One.

[51] Filadi R, Greotti E, Turacchio G, Luini A, Pozzan T, Pizzo P (2015). Mitofusin 2 ablation increases endoplasmic reticulum-mitochondria coupling. Proc Natl Acad Sci U S A.

[52] Naon D, Zaninello M, Giacomello M, Varanita T, Grespi F, Lakshminaranayan S, Serafini A, Semenzato M, Herkenne S, Hernandez-Alvarez MI, et al. Critical reappraisal confirms that Mitofusin 2 is an endoplasmic reticulum-mitochondria tether. Proc Natl Acad Sci U S A. 2016;113(40):11249–54. 10.1073/pnas.1606786113 PMC5056088 27647893

[53] Leal NS, Schreiner B, Pinho CM, Filadi R, Wiehager B, Karlstrom H, Pizzo P, Ankarcrona M (2016). Mitofusin-2 knockdown increases ER-mitochondria contact and decreases amyloid beta-peptide production. J Cell Mol Med.

[54] Filadi R, Greotti E, Turacchio G, Luini A, Pozzan T, Pizzo P (2016). Presenilin 2 modulates endoplasmic reticulum-mitochondria coupling by tuning the antagonistic effect of mitofusin 2. Cell Rep.

[55] Zampese E, Fasolato C, Pozzan T, Pizzo P (2011). Presenilin-2 modulation of ER-mitochondria interactions: FAD mutations, mechanisms and pathological consequences. Commun Integr Biol.

[56] Leboucher GP, Tsai YC, Yang M, Shaw KC, Zhou M, Veenstra TD, Glickman MH, Weissman AM (2012). Stress-induced phosphorylation and proteasomal degradation of mitofusin 2 facilitates mitochondrial fragmentation and apoptosis. Mol Cell.

[57] Mourier A, Motori E, Brandt T, Lagouge M, Atanassov I, Galinier A, Rappl G, Brodesser S, Hultenby K, Dieterich C (2015). Mitofusin 2 is required to maintain mitochondrial coenzyme Q levels. J Cell Biol.

[58] Ding Y, Gao H, Zhao L, Wang X, Zheng M (2015). Mitofusin 2-deficiency suppresses cell proliferation through disturbance of autophagy. PLoS One.

[59] Kawalec M, Boratynska-Jasinska A, Beresewicz M, Dymkowska D, Zablocki K, Zablocka B (2015). Mitofusin 2 deficiency affects energy metabolism and mitochondrial biogenesis in MEF cells. PLoS One.

[60] Wang PT, Garcin PO, Fu M, Masoudi M, St-Pierre P, Pante N, Nabi IR (2015). Distinct mechanisms controlling rough and smooth endoplasmic reticulum contacts with mitochondria. J Cell Sci.

[61] Li L, Gao G, Shankar J, Joshi B, Foster LJ, Nabi IR (2015). p38 MAP kinase-dependent phosphorylation of the Gp78 E3 ubiquitin ligase controls ER-mitochondria association and mitochondria motility. Mol Biol Cell.

[62] Luchsinger LL, de Almeida MJ, Corrigan DJ, Mumau M, Snoeck HW (2016). Mitofusin 2 maintains haematopoietic stem cells with extensive lymphoid potential. Nature.

[63] Bravo R, Vicencio JM, Parra V, Troncoso R, Munoz JP, Bui M, Quiroga C, Rodriguez AE, Verdejo HE, Ferreira J (2011). Increased ER-mitochondrial coupling promotes mitochondrial respiration and bioenergetics during early phases of ER stress. J Cell Sci.

[64] Bravo-Sagua R, Lopez-Crisosto C, Parra V, Rodriguez-Pena M, Rothermel BA, Quest AF, Lavandero S (2016). mTORC1 inhibitor rapamycin and ER stressor tunicamycin induce differential patterns of ER-mitochondria coupling. Sci Rep.

[65] Verfaillie T, Rubio N, Garg AD, Bultynck G, Rizzuto R, Decuypere JP, Piette J, Linehan C, Gupta S, Samali A (2012). PERK is required at the ER-mitochondrial contact sites to convey apoptosis after ROS-based ER stress. Cell Death Differ.

[66] van Vliet AR, Garg AD, Agostinis P (2016). Coordination of stress, Ca2+, and immunogenic signaling pathways by PERK at the endoplasmic reticulum. Biol Chem.

[67] Hermann GJ, Thatcher JW, Mills JP, Hales KG, Fuller MT, Nunnari J, Shaw JM (1998). Mitochondrial fusion in yeast requires the transmembrane GTPase Fzo1p. J Cell Biol.

[68] Rapaport D, Brunner M, Neupert W, Westermann B (1998). Fzo1p is a mitochondrial outer membrane protein essential for the biogenesis of functional mitochondria in Saccharomyces cerevisiae. J Biol Chem.

[69] Harner M, Korner C, Walther D, Mokranjac D, Kaesmacher J, Welsch U, Griffith J, Mann M, Reggiori F, Neupert W (2011). The mitochondrial contact site complex, a determinant of mitochondrial architecture. EMBO J.

[70] Anton F, Fres JM, Schauss A, Pinson B, Praefcke GJ, Langer T, Escobar-Henriques M (2011). Ugo1 and Mdm30 act sequentially during Fzo1-mediated mitochondrial outer membrane fusion. J Cell Sci.

[71] Schumacher MM, Choi JY, Voelker DR (2002). Phosphatidylserine transport to the mitochondria is regulated by ubiquitination. J Biol Chem.

[72] Sugiura A, Nagashima S, Tokuyama T, Amo T, Matsuki Y, Ishido S, Kudo Y, McBride HM, Fukuda T, Matsushita N (2013). MITOL regulates endoplasmic reticulum-mitochondria contacts via Mitofusin2. Mol Cell.

[73] Szabadkai G, Bianchi K, Varnai P, De Stefani D, Wieckowski MR, Cavagna D, Nagy AI, Balla T, Rizzuto R (2006). Chaperone-mediated coupling of endoplasmic reticulum and mitochondrial Ca2+ channels. J Cell Biol.

[74] De Vos KJ, Morotz GM, Stoica R, Tudor EL, Lau KF, Ackerley S, Warley A, Shaw CE, Miller CC (2012). VAPB interacts with the mitochondrial protein PTPIP51 to regulate calcium homeostasis. Hum Mol Genet.

[75] Stoica R, De Vos KJ, Paillusson S, Mueller S, Sancho RM, Lau KF, Vizcay-Barrena G, Lin WL, Xu YF, Lewis J (2014). ER-mitochondria associations are regulated by the VAPB-PTPIP51 interaction and are disrupted by ALS/FTD-associated TDP-43. Nat Commun.

[76] Stoica R, Paillusson S, Gomez-Suaga P, Mitchell JC, Lau DH, Gray EH, Sancho RM, Vizcay-Barrena G, De Vos KJ, Shaw CE (2016). ALS/FTD-associated FUS activates GSK-3beta to disrupt the VAPB-PTPIP51 interaction and ER-mitochondria associations. EMBO Rep.

[77] McMurray WC, Dawson RM (1969). Phospholipid exchange reactions within the liver cell. Biochem J.

[78] Sauner MT, Levy M (1971). Study of the transfer of phospholipids from the endoplasmic reticulum to the outer and inner mitochondrial membranes. J Lipid Res.

[79] Wirtz KW, Zilversmit DB (1968). Exchange of phospholipids between liver mitochondria and microsomes in vitro. J Biol Chem.

[80] Voelker DR (1985). Disruption of phosphatidylserine translocation to the mitochondria in baby hamster kidney cells. J Biol Chem.

[81] Dennis EA, Kennedy EP (1972). Intracellular sites of lipid synthesis and the biogenesis of mitochondria. J Lipid Res.

[82] Cobon GS, Crowfoot PD, Linnane AW (1974). Biogenesis of mitchondria. Phospholipid synthesis in vitro by yeast mitochondrial and microsomal fractions. Biochem J.

[83] Vance JE (1988). Compartmentalization and differential labeling of phospholipids of rat liver subcellular membranes. Biochim Biophys Acta.

[84] Voelker DR (1989). Reconstitution of phosphatidylserine import into rat liver mitochondria. J Biol Chem.

[85] Sugiura A, McLelland GL, Fon EA, McBride HM (2014). A new pathway for mitochondrial quality control: mitochondrial-derived vesicles. EMBO J.

[86] Shiao YJ, Vance JE (1995). Evidence for an ethanolamine cycle: differential recycling of the ethanolamine moiety of phosphatidylethanolamine derived from phosphatidylserine and ethanolamine. Biochem J.

[87] Vance JE (1991). Newly made phosphatidylserine and phosphatidylethanolamine are preferentially translocated between rat liver mitochondria and endoplasmic reticulum. J Biol Chem.

[88] Stone SJ, Levin MC, Zhou P, Han J, Walther TC, Farese RV (2009). The endoplasmic reticulum enzyme DGAT2 is found in mitochondria-associated membranes and has a mitochondrial targeting signal that promotes its association with mitochondria. J Biol Chem.

[89] Shiao YJ, Lupo G, Vance JE (1995). Evidence that phosphatidylserine is imported into mitochondria via a mitochondria-associated membrane and that the majority of mitochondrial phosphatidylethanolamine is derived from decarboxylation of phosphatidylserine. J Biol Chem.

[90] Voelker DR (1989). Phosphatidylserine translocation to the mitochondrion is an ATP-dependent process in permeabilized animal cells. Proc Natl Acad Sci U S A.

[91] Voelker DR (1990). Characterization of phosphatidylserine synthesis and translocation in permeabilized animal cells. J Biol Chem.

[92] Osman C, Voelker DR, Langer T (2011). Making heads or tails of phospholipids in mitochondria. J Cell Biol.

[93] Flis VV, Daum G (2013). Lipid transport between the endoplasmic reticulum and mitochondria. Cold Spring Harb Perspect Biol.

[94] Kuchler K, Daum G, Paltauf F (1986). Subcellular and submitochondrial localization of phospholipid-synthesizing enzymes in Saccharomyces cerevisiae. J Bacteriol.

[95] Zinser E, Sperka-Gottlieb CD, Fasch EV, Kohlwein SD, Paltauf F, Daum G (1991). Phospholipid synthesis and lipid composition of subcellular membranes in the unicellular eukaryote Saccharomyces cerevisiae. J Bacteriol.

[96] Gnamusch E, Kalaus C, Hrastnik C, Paltauf F, Daum G (1992). Transport of phospholipids between subcellular membranes of wild-type yeast cells and of the phosphatidylinositol transfer protein-deficient strain Saccharomyces cerevisiae sec 14. Biochim Biophys Acta.

[97] Simbeni R, Tangemann K, Schmidt M, Ceolotto C, Paltauf F, Daum G (1993). Import of phosphatidylserine into isolated yeast mitochondria. Biochim Biophys Acta.

[98] Trotter PJ, Pedretti J, Voelker DR (1993). Phosphatidylserine decarboxylase from Saccharomyces cerevisiae. Isolation of mutants, cloning of the gene, and creation of a null allele. J Biol Chem.

[99] Trotter PJ, Voelker DR (1995). Identification of a non-mitochondrial phosphatidylserine decarboxylase activity (PSD2) in the yeast Saccharomyces cerevisiae. J Biol Chem.

[100] Cui Z, Vance JE, Chen MH, Voelker DR, Vance DE (1993). Cloning and expression of a novel phosphatidylethanolamine N-methyltransferase. A specific biochemical and cytological marker for a unique membrane fraction in rat liver. J Biol Chem.

[101] Sakakibara K, Eiyama A, Suzuki SW, Sakoh-Nakatogawa M, Okumura N, Tani M, Hashimoto A, Nagumo S, Kondo-Okamoto N, Kondo-Kakuta C (2015). Phospholipid methylation controls Atg32-mediated mitophagy and Atg8 recycling. EMBO J.

[102] Claypool SM, Koehler CM (2012). The complexity of cardiolipin in health and disease. Trends Biochem Sci.

[103] Schlattner U, Tokarska-Schlattner M, Rousseau D, Boissan M, Mannella C, Epand R, Lacombe ML (2014). Mitochondrial cardiolipin/phospholipid trafficking: the role of membrane contact site complexes and lipid transfer proteins. Chem Phys Lipids.

[104] Connerth M, Tatsuta T, Haag M, Klecker T, Westermann B, Langer T (2012). Intramitochondrial transport of phosphatidic acid in yeast by a lipid transfer protein. Science.

[105] Abrams AJ, Hufnagel RB, Rebelo A, Zanna C, Patel N, Gonzalez MA, Campeanu IJ, Griffin LB, Groenewald S, Strickland AV (2015). Mutations in SLC25A46, encoding a UGO1-like protein, cause an optic atrophy spectrum disorder. Nat Genet.

[106] Adachi Y, Itoh K, Yamada T, Cerveny KL, Suzuki TL, Macdonald P, Frohman MA, Ramachandran R, Iijima M, Sesaki H (2016). Coincident phosphatidic acid interaction restrains Drp1 in mitochondrial division. Mol Cell.

[107] Porter TD (2015). Electron transfer pathways in cholesterol synthesis. Lipids.

[108] Lucken-Ardjomande S, Montessuit S, Martinou JC (2008). Bax activation and stress-induced apoptosis delayed by the accumulation of cholesterol in mitochondrial membranes. Cell Death Differ.

[109] Altmann K, Westermann B (2005). Role of essential genes in mitochondrial morphogenesis in Saccharomyces cerevisiae. Mol Biol Cell.

[110] Miller WL (2013). Steroid hormone synthesis in mitochondria. Mol Cell Endocrinol.

[111] Bose HS, Lingappa VR, Miller WL (2002). Rapid regulation of steroidogenesis by mitochondrial protein import. Nature.

[112] Campbell AM, Chan SH (2007). The voltage dependent anion channel affects mitochondrial cholesterol distribution and function. Arch Biochem Biophys.

[113] Gatta AT, Wong LH, Sere YY, Calderon-Norena DM, Cockcroft S, Menon AK, Levine TP. A new family of StART domain proteins at membrane contact sites has a role in ER-PM sterol transport. Elife 2015;4 10.7554/eLife.07253 PMC4463742 26001273

[114] Murley A, Sarsam RD, Toulmay A, Yamada J, Prinz WA, Nunnari J (2015). Ltc1 is an ER-localized sterol transporter and a component of ER-mitochondria and ER-vacuole contacts. J Cell Biol.

[115] Elbaz-Alon Y, Eisenberg-Bord M, Shinder V, Stiller SB, Shimoni E, Wiedemann N, Geiger T, Schuldiner M (2015). Lam6 regulates the extent of contacts between organelles. Cell Rep.

[116] Area-Gomez E, Del Carmen Lara Castillo M, Tambini MD, Guardia-Laguarta C, De Groof AJ, Madra M, Ikenouchi J, Umeda M, Bird TD, Sturley SL (2012). Upregulated function of mitochondria-associated ER membranes in Alzheimer disease. EMBO J.

[117] Fujimoto M, Hayashi T, Su TP (2012). The role of cholesterol in the association of endoplasmic reticulum membranes with mitochondria. Biochem Biophys Res Commun.

[118] Hayashi T, Fujimoto M (2010). Detergent-resistant microdomains determine the localization of sigma-1 receptors to the endoplasmic reticulum-mitochondria junction. Mol Pharmacol.

[119] Williamson CD, Zhang A, Colberg-Poley AM (2011). The human cytomegalovirus protein UL37 exon 1 associates with internal lipid rafts. J Virol.

[120] Bosch M, Mari M, Gross SP, Fernandez-Checa JC, Pol A (2011). Mitochondrial cholesterol: a connection between caveolin, metabolism, and disease. Traffic.

[121] Bosch M, Mari M, Herms A, Fernandez A, Fajardo A, Kassan A, Giralt A, Colell A, Balgoma D, Barbero E (2011). Caveolin-1 deficiency causes cholesterol-dependent mitochondrial dysfunction and apoptotic susceptibility. Curr Biol.

[122] Sano R, Annunziata I, Patterson A, Moshiach S, Gomero E, Opferman J, Forte M, d’Azzo A (2009). GM1-ganglioside accumulation at the mitochondria-associated ER membranes links ER stress to Ca(2+)-dependent mitochondrial apoptosis. Mol Cell.

[123] Zheng YZ, Berg KB, Foster LJ (2009). Mitochondria do not contain lipid rafts, and lipid rafts do not contain mitochondrial proteins. J Lipid Res.

[124] Sala-Vila A, Navarro-Lerida I, Sanchez-Alvarez M, Bosch M, Calvo C, Lopez JA, Calvo E, Ferguson C, Giacomello M, Serafini A (2016). Interplay between hepatic mitochondria-associated membranes, lipid metabolism and caveolin-1 in mice. Sci Rep.

[125] Ciarlo L, Manganelli V, Garofalo T, Matarrese P, Tinari A, Misasi R, Malorni W, Sorice M (2010). Association of fission proteins with mitochondrial raft-like domains. Cell Death Differ.

[126] Ziolkowski W, Szkatula M, Nurczyk A, Wakabayashi T, Kaczor JJ, Olek RA, Knap N, Antosiewicz J, Wieckowski MR, Wozniak M (2010). Methyl-beta-cyclodextrin induces mitochondrial cholesterol depletion and alters the mitochondrial structure and bioenergetics. FEBS Lett.

[127] Du X, Brown AJ, Yang H (2015). Novel mechanisms of intracellular cholesterol transport: oxysterol-binding proteins and membrane contact sites. Curr Opin Cell Biol.

[128] Loewen CJ, Roy A, Levine TP (2003). A conserved ER targeting motif in three families of lipid binding proteins and in Opi1p binds VAP. EMBO J.

[129] Drin G, von Filseck JM, Copic A (2016). New molecular mechanisms of inter-organelle lipid transport. Biochem Soc Trans.

[130] Galmes R, Houcine A, van Vliet AR, Agostinis P, Jackson CL, Giordano F. ORP5/ORP8 localize to endoplasmic reticulum-mitochondria contacts and are involved in mitochondrial function. EMBO Rep. 2016;17(6):800–10. doi:10.15252/embr.201541108. 10.15252/embr.201541108 PMC5278607 27113756

[131] Gellerich FN, Gizatullina Z, Trumbeckaite S, Nguyen HP, Pallas T, Arandarcikaite O, Vielhaber S, Seppet E, Striggow F (2010). The regulation of OXPHOS by extramitochondrial calcium. Biochim Biophys Acta.

[132] Denton RM (2009). Regulation of mitochondrial dehydrogenases by calcium ions. Biochim Biophys Acta.

[133] Tasseva G, Bai HD, Davidescu M, Haromy A, Michelakis E, Vance JE (2013). Phosphatidylethanolamine deficiency in Mammalian mitochondria impairs oxidative phosphorylation and alters mitochondrial morphology. J Biol Chem.

[134] Rohacs T, Tory K, Dobos A, Spat A (1997). Intracellular calcium release is more efficient than calcium influx in stimulating mitochondrial NAD(P)H formation in adrenal glomerulosa cells. Biochem J.

[135] Hurst S, Hoek J, Sheu SS. Mitochondrial Ca2+ and regulation of the permeability transition pore. J Bioenerg Biomembr. 2016. In press. 10.1007/s10863-016-9672-x PMC5393273 27497945

[136] Hunter DR, Haworth RA, Southard JH (1976). Relationship between configuration, function, and permeability in calcium-treated mitochondria. J Biol Chem.

[137] Capponi AM, Rossier MF, Davies E, Vallotton MB (1988). Calcium stimulates steroidogenesis in permeabilized bovine adrenal cortical cells. J Biol Chem.

[138] Cherradi N, Rossier MF, Vallotton MB, Capponi AM (1996). Calcium stimulates intramitochondrial cholesterol transfer in bovine adrenal glomerulosa cells. J Biol Chem.

[139] Yi M, Weaver D, Hajnoczky G (2004). Control of mitochondrial motility and distribution by the calcium signal: a homeostatic circuit. J Cell Biol.

[140] MacAskill AF, Brickley K, Stephenson FA, Kittler JT (2009). GTPase dependent recruitment of Grif-1 by Miro1 regulates mitochondrial trafficking in hippocampal neurons. Mol Cell Neurosci.

[141] Saotome M, Safiulina D, Szabadkai G, Das S, Fransson A, Aspenstrom P, Rizzuto R, Hajnoczky G (2008). Bidirectional Ca2 + −dependent control of mitochondrial dynamics by the Miro GTPase. Proc Natl Acad Sci U S A.

[142] Wang X, Schwarz TL (2009). The mechanism of Ca2 + − dependent regulation of kinesin-mediated mitochondrial motility. Cell.

[143] Bravo R, Parra V, Gatica D, Rodriguez AE, Torrealba N, Paredes F, Wang ZV, Zorzano A, Hill JA, Jaimovich E (2013). Endoplasmic reticulum and the unfolded protein response: dynamics and metabolic integration. Int Rev Cell Mol Biol.

[144] Jonikas MC, Collins SR, Denic V, Oh E, Quan EM, Schmid V, Weibezahn J, Schwappach B, Walter P, Weissman JS (2009). Comprehensive characterization of genes required for protein folding in the endoplasmic reticulum. Science.

[145] Carafoli E, Balcavage WX, Lehninger AL, Mattoon JR (1970). Ca2+ metabolism in yeast cells and mitochondria. Biochim Biophys Acta.

[146] Biden TJ, Wollheim CB, Schlegel W (1986). Inositol 1,4,5-trisphosphate and intracellular Ca2+ homeostasis in clonal pituitary cells (GH3). Translocation of Ca2+ into mitochondria from a functionally discrete portion of the nonmitochondrial store. J Biol Chem.

[147] Halachmi D, Eilam Y (1989). Cytosolic and vacuolar Ca2+ concentrations in yeast cells measured with the Ca2 + −sensitive fluorescence dye indo-1. FEBS Lett.

[148] Baughman JM, Perocchi F, Girgis HS, Plovanich M, Belcher-Timme CA, Sancak Y, Bao XR, Strittmatter L, Goldberger O, Bogorad RL (2011). Integrative genomics identifies MCU as an essential component of the mitochondrial calcium uniporter. Nature.

[149] De Stefani D, Raffaello A, Teardo E, Szabo I, Rizzuto R (2011). A forty-kilodalton protein of the inner membrane is the mitochondrial calcium uniporter. Nature.

[150] Kovacs-Bogdan E, Sancak Y, Kamer KJ, Plovanich M, Jambhekar A, Huber RJ, Myre MA, Blower MD, Mootha VK (2014). Reconstitution of the mitochondrial calcium uniporter in yeast. Proc Natl Acad Sci U S A.

[151] Uribe S, Rangel P, Pardo JP (1992). Interactions of calcium with yeast mitochondria. Cell Calcium.

[152] Bradshaw PC, Jung DW, Pfeiffer DR (2001). Free fatty acids activate a vigorous Ca(2+):2H(+) antiport activity in yeast mitochondria. J Biol Chem.

[153] Bazhenova EN, Saris NE, Zvyagilskaya RA (1998). Stimulation of the yeast mitochondrial calcium uniporter by hypotonicity and by ruthenium red. Biochim Biophys Acta.

[154] Carraro M, Bernardi P (2016). Calcium and reactive oxygen species in regulation of the mitochondrial permeability transition and of programmed cell death in yeast. Cell Calcium.

[155] Becker GL, Fiskum G, Lehninger AL (1980). Regulation of free Ca2+ by liver mitochondria and endoplasmic reticulum. J Biol Chem.

[156] Meier PJ, Spycher MA, Meyer UA (1981). Isolation and characterization of rough endoplasmic reticulum associated with mitochondria from normal rat liver. Biochim Biophys Acta.

[157] Csordas G, Thomas AP, Hajnoczky G (1999). Quasi-synaptic calcium signal transmission between endoplasmic reticulum and mitochondria. Embo J.

[158] Csordas G, Varnai P, Golenar T, Roy S, Purkins G, Schneider TG, Balla T, Hajnoczky G (2010). Imaging interorganelle contacts and local calcium dynamics at the ER-mitochondrial interface. Mol Cell.

[159] Rizzuto R, Pinton P, Carrington W, Fay FS, Fogarty KE, Lifshitz LM, Tuft RA, Pozzan T (1998). Close contacts with the endoplasmic reticulum as determinants of mitochondrial Ca2+ responses. Science.

[160] Rizzuto R, Brini M, Murgia M, Pozzan T (1993). Microdomains with high Ca2+ close to IP3-sensitive channels that are sensed by neighboring mitochondria. Science.

[161] Mallilankaraman K, Doonan P, Cardenas C, Chandramoorthy HC, Muller M, Miller R, Hoffman NE, Gandhirajan RK, Molgo J, Birnbaum MJ (2012). MICU1 is an essential gatekeeper for MCU-mediated mitochondrial Ca(2+) uptake that regulates cell survival. Cell.

[162] Perocchi F, Gohil VM, Girgis HS, Bao XR, McCombs JE, Palmer AE, Mootha VK (2010). MICU1 encodes a mitochondrial EF hand protein required for Ca(2+) uptake. Nature.

[163] Plovanich M, Bogorad RL, Sancak Y, Kamer KJ, Strittmatter L, Li AA, Girgis HS, Kuchimanchi S, De Groot J, Speciner L (2013). MICU2, a paralog of MICU1, resides within the mitochondrial uniporter complex to regulate calcium handling. PLoS One.

[164] Jouaville LS, Pinton P, Bastianutto C, Rutter GA, Rizzuto R (1999). Regulation of mitochondrial ATP synthesis by calcium: evidence for a long-term metabolic priming. Proc Natl Acad Sci U S A.

[165] Cardenas C, Miller RA, Smith I, Bui T, Molgo J, Muller M, Vais H, Cheung KH, Yang J, Parker I (2010). Essential regulation of cell bioenergetics by constitutive InsP3 receptor Ca2+ transfer to mitochondria. Cell.

[166] Pan X, Liu J, Nguyen T, Liu C, Sun J, Teng Y, Fergusson MM, Rovira II, Allen M, Springer DA (2013). The physiological role of mitochondrial calcium revealed by mice lacking the mitochondrial calcium uniporter. Nat Cell Biol.

[167] Yamada A, Yamamoto T, Yoshimura Y, Gouda S, Kawashima S, Yamazaki N, Yamashita K, Kataoka M, Nagata T, Terada H (2009). Ca2 + −induced permeability transition can be observed even in yeast mitochondria under optimized experimental conditions. Biochim Biophys Acta.

[168] Perez-Vazquez V, Saavedra-Molina A, Uribe S (2003). In Saccharomyces cerevisiae, cations control the fate of the energy derived from oxidative metabolism through the opening and closing of the yeast mitochondrial unselective channel. J Bioenerg Biomembr.

[169] Mayinger P, Bankaitis VA, Meyer DI (1995). Sac1p mediates the adenosine triphosphate transport into yeast endoplasmic reticulum that is required for protein translocation. J Cell Biol.

[170] Moser von Filseck J, Copic A, Delfosse V, Vanni S, Jackson CL, Bourguet W, Drin G (2015). INTRACELLULAR TRANSPORT. Phosphatidylserine transport by ORP/Osh proteins is driven by phosphatidylinositol 4-phosphate. Science.

[171] Moser von Filseck J, Vanni S, Mesmin B, Antonny B, Drin G (2015). A phosphatidylinositol-4-phosphate powered exchange mechanism to create a lipid gradient between membranes. Nat Commun.

[172] Blagoveshchenskaya A, Cheong FY, Rohde HM, Glover G, Knodler A, Nicolson T, Boehmelt G, Mayinger P (2008). Integration of Golgi trafficking and growth factor signaling by the lipid phosphatase SAC1. J Cell Biol.

[173] Simmen T, Lynes EM, Gesson K, Thomas G (2010). Oxidative protein folding in the endoplasmic reticulum: tight links to the mitochondria-associated membrane (MAM). Biochim Biophys Acta.

[174] Camacho P, Lechleiter JD (1993). Increased frequency of calcium waves in Xenopus laevis oocytes that express a calcium-ATPase. Science.

[175] Jouaville LS, Ichas F, Holmuhamedov EL, Camacho P, Lechleiter JD (1995). Synchronization of calcium waves by mitochondrial substrates in Xenopus laevis oocytes. Nature.

[176] Walsh C, Barrow S, Voronina S, Chvanov M, Petersen OH, Tepikin A (2009). Modulation of calcium signalling by mitochondria. Biochim Biophys Acta.

[177] Booth DM, Enyedi B, Geiszt M, Varnai P, Hajnoczky G (2016). Redox nanodomains are induced by and control calcium signaling at the ER-mitochondrial interface. Mol Cell.

[178] Raturi A, Ortiz-Sandoval C, Simmen T (2014). Redox dependence of endoplasmic reticulum (ER) Ca(2)(+) signaling. Histol Histopathol.

[179] John LM, Lechleiter JD, Camacho P (1998). Differential modulation of SERCA2 isoforms by calreticulin. J Cell Biol.

[180] Roderick HL, Lechleiter JD, Camacho P (2000). Cytosolic phosphorylation of calnexin controls intracellular Ca(2+) oscillations via an interaction with SERCA2b. J Cell Biol.

[181] Li Y, Camacho P (2004). Ca2 + −dependent redox modulation of SERCA 2b by ERp57. J Cell Biol.

[182] Lynes EM, Raturi A, Shenkman M, Ortiz Sandoval C, Yap MC, Wu J, Janowicz A, Myhill N, Benson MD, Campbell RE (2013). Palmitoylation is the switch that assigns calnexin to quality control or ER Ca2+ signaling. J Cell Sci.

[183] Bui M, Gilady SY, Fitzsimmons RE, Benson MD, Lynes EM, Gesson K, Alto NM, Strack S, Scott JD, Simmen T (2010). Rab32 modulates apoptosis onset and mitochondria-associated membrane (MAM) properties. J Biol Chem.

[184] Ortiz-Sandoval CG, Hughes SC, Dacks JB, Simmen T (2014). Interaction with the effector dynamin-related protein 1 (Drp1) is an ancient function of Rab32 subfamily proteins. Cell Logist.

[185] Arnaudeau S, Kelley WL, Walsh JV, Demaurex N (2001). Mitochondria recycle Ca(2+) to the endoplasmic reticulum and prevent the depletion of neighboring endoplasmic reticulum regions. J Biol Chem.

[186] Raturi A, Gutierrez T, Ortiz-Sandoval C, Ruangkittisakul A, Herrera-Cruz MS, Rockley JP, Gesson K, Ourdev D, Lou PH, Lucchinetti E (2016). TMX1 determines cancer cell metabolism as a thiol-based modulator of ER-mitochondria Ca2+ flux. J Cell Biol.

[187] Li G, Mongillo M, Chin KT, Harding H, Ron D, Marks AR, Tabas I (2009). Role of ERO1-alpha-mediated stimulation of inositol 1,4,5-triphosphate receptor activity in endoplasmic reticulum stress-induced apoptosis. J Cell Biol.

[188] Gilady SY, Bui M, Lynes EM, Benson MD, Watts R, Vance JE, Simmen T (2010). Ero1alpha requires oxidizing and normoxic conditions to localize to the mitochondria-associated membrane (MAM). Cell Stress Chaperones.

[189] Groenendyk J, Zuppini A, Shore G, Opas M, Bleackley RC, Michalak M (2006). Caspase 12 in calnexin-deficient cells. Biochemistry.

[190] Guerin R, Beauregard PB, Leroux A, Rokeach LA (2009). Calnexin regulates apoptosis induced by inositol starvation in fission yeast. PLoS One.

[191] Boehning D, Patterson RL, Sedaghat L, Glebova NO, Kurosaki T, Snyder SH (2003). Cytochrome c binds to inositol (1,4,5) trisphosphate receptors, amplifying calcium-dependent apoptosis. Nat Cell Biol.

[192] Rigamonti M, Groppi S, Belotti F, Ambrosini R, Filippi G, Martegani E, Tisi R (2015). Hypotonic stress-induced calcium signaling in Saccharomyces cerevisiae involves TRP-like transporters on the endoplasmic reticulum membrane. Cell Calcium.

[193] Severin FF, Hyman AA (2002). Pheromone induces programmed cell death in S. cerevisiae. Curr Biol.

[194] Pozniakovsky AI, Knorre DA, Markova OV, Hyman AA, Skulachev VP, Severin FF (2005). Role of mitochondria in the pheromone- and amiodarone-induced programmed death of yeast. J Cell Biol.

[195] Guaragnella N, Zdralevic M, Antonacci L, Passarella S, Marra E, Giannattasio S (2012). The role of mitochondria in yeast programmed cell death. Front Oncol.

[196] Vervliet T, Parys JB, Bultynck G. Bcl-2 proteins and calcium signaling: complexity beneath the surface. Oncogene. 2016;35(39):5079–92. 10.1038/onc.2016.31 26973249

[197] Yang J, Vais H, Gu W, Foskett JK (2016). Biphasic regulation of InsP3 receptor gating by dual Ca2+ release channel BH3-like domains mediates Bcl-xL control of cell viability. Proc Natl Acad Sci U S A.

[198] Williams A, Hayashi T, Wolozny D, Yin B, Su TC, Betenbaugh MJ, Su TP (2016). The non-apoptotic action of Bcl-xL: regulating Ca(2+) signaling and bioenergetics at the ER-mitochondrion interface. J Bioenerg Biomembr.

[199] Chen R, Valencia I, Zhong F, McColl KS, Roderick HL, Bootman MD, Berridge MJ, Conway SJ, Holmes AB, Mignery GA (2004). Bcl-2 functionally interacts with inositol 1,4,5-trisphosphate receptors to regulate calcium release from the ER in response to inositol 1,4,5-trisphosphate. J Cell Biol.

[200] Hanson CJ, Bootman MD, Distelhorst CW, Wojcikiewicz RJ, Roderick HL (2008). Bcl-2 suppresses Ca2+ release through inositol 1,4,5-trisphosphate receptors and inhibits Ca2+ uptake by mitochondria without affecting ER calcium store content. Cell Calcium.

[201] Monaco G, Decrock E, Akl H, Ponsaerts R, Vervliet T, Luyten T, De Maeyer M, Missiaen L, Distelhorst CW, De Smedt H (2012). Selective regulation of IP3-receptor-mediated Ca2+ signaling and apoptosis by the BH4 domain of Bcl-2 versus Bcl-Xl. Cell Death Differ.

[202] Rong YP, Bultynck G, Aromolaran AS, Zhong F, Parys JB, De Smedt H, Mignery GA, Roderick HL, Bootman MD, Distelhorst CW (2009). The BH4 domain of Bcl-2 inhibits ER calcium release and apoptosis by binding the regulatory and coupling domain of the IP3 receptor. Proc Natl Acad Sci U S A.

[203] Monaco G, Decrock E, Arbel N, van Vliet AR, La Rovere RM, De Smedt H, Parys JB, Agostinis P, Leybaert L, Shoshan-Barmatz V (2015). The BH4 domain of anti-apoptotic Bcl-XL, but not that of the related Bcl-2, limits the voltage-dependent anion channel 1 (VDAC1)-mediated transfer of pro-apoptotic Ca2+ signals to mitochondria. J Biol Chem.

[204] Buttner S, Ruli D, Vogtle FN, Galluzzi L, Moitzi B, Eisenberg T, Kepp O, Habernig L, Carmona-Gutierrez D, Rockenfeller P (2011). A yeast BH3-only protein mediates the mitochondrial pathway of apoptosis. EMBO J.

[205] Plattner H, Verkhratsky A (2016). Inseparable tandem: evolution chooses ATP and Ca2+ to control life, death and cellular signalling. Philos Trans R Soc Lond B Biol Sci.

[206] Lee JE, Westrate LM, Wu H, Page C, Voeltz GK (2016). Multiple dynamin family members collaborate to drive mitochondrial division. Nature.

[207] Lackner LL, Ping H, Graef M, Murley A, Nunnari J (2013). Endoplasmic reticulum-associated mitochondria-cortex tether functions in the distribution and inheritance of mitochondria. Proc Natl Acad Sci U S A.

[208] Wilson JD, Barlowe C (2010). Yet1p and Yet3p, the yeast homologs of BAP29 and BAP31, interact with the endoplasmic reticulum translocation apparatus and are required for inositol prototrophy. J Biol Chem.

[209] Wilson JD, Thompson SL, Barlowe C (2011). Yet1p-Yet3p interacts with Scs2p-Opi1p to regulate ER localization of the Opi1p repressor. Mol Biol Cell.

[210] Gerhold JM, Cansiz-Arda S, Lohmus M, Engberg O, Reyes A, van Rennes H, Sanz A, Holt IJ, Cooper HM, Spelbrink JN (2015). Human mitochondrial DNA-protein complexes attach to a cholesterol-rich membrane structure. Sci Rep.

[211] Shen Y, Ng LF, Low NP, Hagen T, Gruber J, Inoue T (2016). C. Elegans miro-1 mutation reduces the amount of mitochondria and extends life span. PLoS One.

[212] Shibutani ST, Yoshimori T (2014). A current perspective of autophagosome biogenesis. Cell Res.

[213] De Duve C, Wattiaux R (1966). Functions of lysosomes. Annu Rev Physiol.

[214] Novikoff AB, Shin WY (1978). Endoplasmic reticulum and autophagy in rat hepatocytes. Proc Natl Acad Sci U S A.

[215] Rubinsztein DC, Shpilka T, Elazar Z (2012). Mechanisms of autophagosome biogenesis. Curr Biol.

[216] Axe EL, Walker SA, Manifava M, Chandra P, Roderick HL, Habermann A, Griffiths G, Ktistakis NT (2008). Autophagosome formation from membrane compartments enriched in phosphatidylinositol 3-phosphate and dynamically connected to the endoplasmic reticulum. J Cell Biol.

[217] Suzuki K, Kirisako T, Kamada Y, Mizushima N, Noda T, Ohsumi Y (2001). The pre-autophagosomal structure organized by concerted functions of APG genes is essential for autophagosome formation. EMBO J.

[218] Itakura E, Mizushima N (2010). Characterization of autophagosome formation site by a hierarchical analysis of mammalian Atg proteins. Autophagy.

[219] Garofalo T, Matarrese P, Manganelli V, Marconi M, Tinari A, Gambardella L, Faggioni A, Misasi R, Sorice M, Malorni W (2016). Evidence for the involvement of lipid rafts localized at the ER-mitochondria associated membranes in autophagosome formation. Autophagy.

[220] Wu W, Lin C, Wu K, Jiang L, Wang X, Li W, Zhuang H, Zhang X, Chen H, Li S (2016). FUNDC1 regulates mitochondrial dynamics at the ER-mitochondrial contact site under hypoxic conditions. EMBO J.

[221] Hailey DW, Rambold AS, Satpute-Krishnan P, Mitra K, Sougrat R, Kim PK, Lippincott-Schwartz J (2010). Mitochondria supply membranes for autophagosome biogenesis during starvation. Cell.

[222] Itakura E, Kishi-Itakura C, Mizushima N (2012). The hairpin-type tail-anchored SNARE syntaxin 17 targets to autophagosomes for fusion with endosomes/lysosomes. Cell.

[223] Arasaki K, Shimizu H, Mogari H, Nishida N, Hirota N, Furuno A, Kudo Y, Baba M, Baba N, Cheng J (2015). A role for the ancient SNARE syntaxin 17 in regulating mitochondrial division. Dev Cell.

[224] Bockler S, Westermann B (2014). Mitochondrial ER contacts are crucial for mitophagy in yeast. Dev Cell.

[225] Khaminets A, Heinrich T, Mari M, Grumati P, Huebner AK, Akutsu M, Liebmann L, Stolz A, Nietzsche S, Koch N (2015). Regulation of endoplasmic reticulum turnover by selective autophagy. Nature.

[226] Mochida K, Oikawa Y, Kimura Y, Kirisako H, Hirano H, Ohsumi Y, Nakatogawa H (2015). Receptor-mediated selective autophagy degrades the endoplasmic reticulum and the nucleus. Nature.

[227] Krols M, van Isterdael G, Asselbergh B, Kremer A, Lippens S, Timmerman V, Janssens S (2016). Mitochondria-associated membranes as hubs for neurodegeneration. Acta Neuropathol.

[228] Patergnani S, Missiroli S, Marchi S, Giorgi C (2015). Mitochondria-associated endoplasmic reticulum membranes microenvironment: targeting autophagic and apoptotic pathways in cancer therapy. Front Oncol.

[229] Rutter GA, Pinton P (2014). Mitochondria-associated endoplasmic reticulum membranes in insulin signaling. Diabetes.

[230] Lopez-Crisosto C, Bravo-Sagua R, Rodriguez-Pena M, Mera C, Castro PF, Quest AF, Rothermel BA, Cifuentes M, Lavandero S (2015). ER-to-mitochondria miscommunication and metabolic diseases. Biochim Biophys Acta.

